# N-glycomics profiling reveals alteration of fucosylation in early acute ischemic stroke from mouse brain tissue to human serum

**DOI:** 10.1186/s12014-025-09578-w

**Published:** 2026-01-28

**Authors:** Yike Wu, Linghui Hu, Jianlin Huang, Yunxue Zhong, Kangcheng Li, Zhou Qiu, Li Su, Yuan Zhang, Wenlan Liu

**Affiliations:** 1 Department of Laboratory Medicine, Shenzhen Institute of Translational Medicine, Shenzhen Second People’s Hospital, The First Affiliated Hospital of Shenzhen University, Shenzhen, 518035 China; 2 Department of Neurosurgery, Shenzhen Second People’s Hospital, The First Affiliated Hospital of Shenzhen University, Shenzhen, 518035 China; 3 Department of Neurosurgery, South China Hospital Affiliated to Shenzhen University, Shenzhen, 518055 China; 4 Medical Genetics Center, Shenzhen Maternity & Child Healthcare Hospital, Shenzhen, 518028 China; 5 ShenZhen Key Laboratory of Neurosurgery, Shenzhen Second People’s Hospital, the First Affiliated Hospital of Shenzhen University, 518035 Shenzhen, China

**Keywords:** N-glycome, Early AIS, Glyco-biomarkers, Fucosylation

## Abstract

**Background:**

Accumulating evidence suggests that N-glycosylation plays a crucial role in modulating ischemic pathophysiology. However, the dynamic alterations of N-glycosylation patterns during the early phase of acute ischemic stroke (AIS) have not been systematically investigated.

**Methods:**

We employed matrix assisted laser desorption ionization time-of-flight mass spectrometry (MALDI-TOF-MS) to profile the glycome of murine cerebral tissues and identify differentially expressed glycans and glycosylation features during early AIS progression. To validate these findings, we further analyzed serum glycome profiles from human AIS patients in the early disease stage. Comprehensive statistical analyses were conducted to identify potential glycome biomarkers.

**Results:**

Comprehensive glycomic profiling identified 42 distinct N-glycan structures in murine brain tissues and 31 in serum samples. Through integrated multivariate statistical analyses, we identified 9 ischemia-sensitive cerebral glycans and 6 serum biomarkers. While no individual glycan structures were conserved between species, we identified a conserved downregulation of fucosylation as a key feature (murine cohort AUC = 0.98; human cohort AUC = 0.78).

**Conclusion:**

This study presents the first comprehensive comparison of glycomic profiles in early AIS from mouse brain tissue and human serum, identifying a conserved fucosylation deficiency as a potential class of diagnostic indicators for clinical detection and therapeutic targeting. The observed deficiency was correlated with a potential mechanistic link to dysregulated glycan-mediated inflammatory responses and impaired cellular stress response pathways.

**Graphical abstract:**

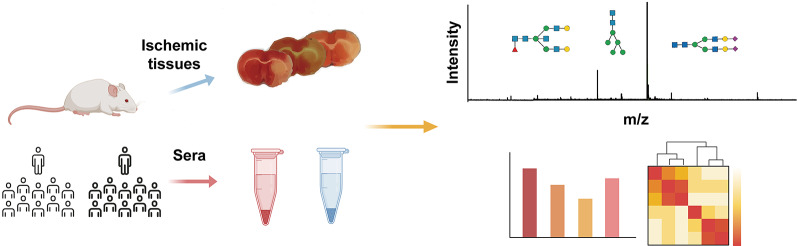

**Supplementary Information:**

The online version contains supplementary material available at 10.1186/s12014-025-09578-w.

## Introduction

Acute ischemic stroke (AIS) presents a critical global health challenge characterized by substantial morbidity, mortality, disability, and recurrence rates, imposing severe socioeconomic burdens on healthcare systems worldwide [1]. While revascularization therapy remains the gold-standard intervention, its clinical utility is significantly constrained by a narrow therapeutic window (within 6 h post-onset) and variable patient responses [2]. The time-dependent nature of neuronal apoptosis following cerebral hypoperfusion underscores the critical need for both rapid diagnostic confirmation and accurate prediction of treatment outcomes. Current clinical practice lacks reliable blood biomarkers capable of simultaneously evaluating two crucial parameters: (1) revascularization efficacy through ischemic penumbra quantification, and (2) hemorrhagic transformation risk via blood-brain barrier (BBB) integrity assessment. This diagnostic gap highlights the urgent requirement for novel circulating biomarkers that enable precision medicine approaches in AIS management.

Contemporary biomarker research pursues two primary objectives: (1) development of rapid diagnostic tools for early stroke detection, and (2) creation of predictive models to guide individualized therapeutic strategies [3, 4]. Recent advances in glycobiology have revealed protein N-glycosylation-a ubiquitous post-translational modification regulating protein localization, cellular interactions, and signal transduction - as a promising biomarker source [5]. The emerging field of N-glycomics demonstrates particular diagnostic potential through its ability to detect disease-specific glycosylation patterns in accessible biofluids, offering advantages in cost-effectiveness and clinical feasibility [6, 7]. Mechanistic studies have established crucial links between aberrant N-glycosylation profiles and AIS pathophysiology, including inflammatory modulation, neuronal apoptosis, cerebrovascular dysfunction, and ischemic injury progression [8–11]. Despite these advances, the temporal dynamics of N-glycan alterations during early AIS pathogenesis remain poorly characterized, representing a critical knowledge gap in stroke biomarker development.

**Fig. 1:**
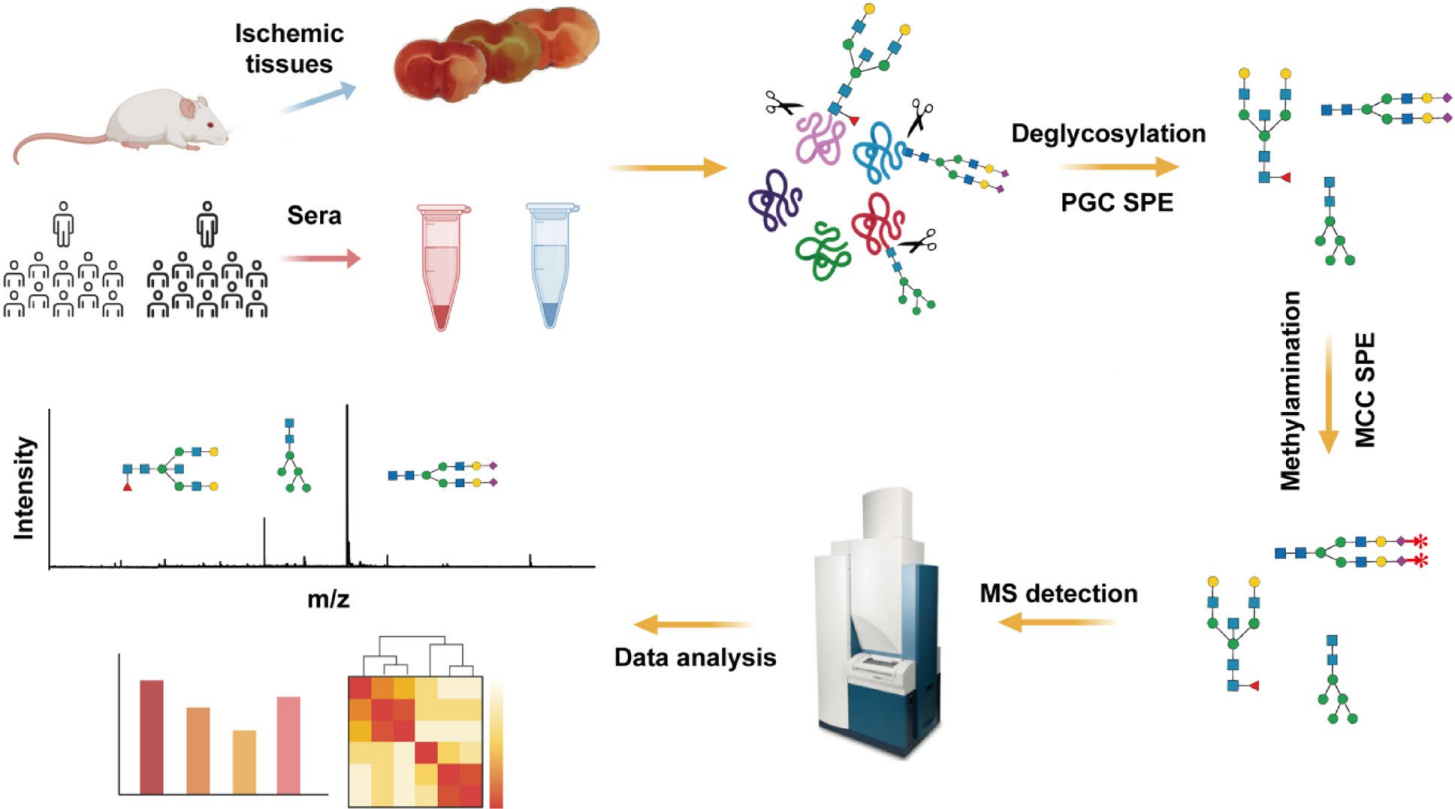
Analysis process of N-glycomic profiles in ischemic brain tissues from middle cerebral artery occlusion (MCAO) mice or sera from acute ischemic stroke (AIS) patients. The workflow consists of protein extraction, N-glycans release, derivatization, purification, MS detection and profiles analysis

This investigation employs a translational research paradigm to address this unmet need. Using a murine middle cerebral artery occlusion model, we first conducted longitudinal N-glycomic profiling of ischemic brain tissue, identifying significant sialylation and fucosylation modifications within 3 h post-ischemia. Subsequent clinical validation revealed conserved fucosylation patterns in serum samples from AIS patients compared to healthy controls, demonstrating remarkable cross-species consistency. Our findings establish fucosylated N-glycans as promising candidate biomarkers for early AIS detection, with particular clinical relevance given their non-invasive accessibility in peripheral blood. The workflow for protein extraction, enzymatic release of N-glycans, derivatization, and MS analyses was in accordance with the international guideline MIRAGE (Minimum Information Required for a Glycomics Experiment) [12] and illustrated in Fig. 1.

## Materials and methods

### Chemicals and reagents

The N-glycans release kit including peptide-N-glycosidase F (PNGase F, 500,000 units/mL), glycobuffer (10x), glycoprotein denaturing buffer (10x) and 10% NP-40 was purchased from New England Biolabs (Ipswich, MA). Acetonitrile (ACN), butanol, ethanol, formic acid (FA) and PBS (pH 7.4, 10 mM) were purchased from Thermo Fisher Scientific (Fair Lawn, NJ). Super-2,5-Dihydroxy benzoic acid (DHB), trifluoroacetic acid (TFA), dimethyl sulfoxide (DMSO), methylamine hydrochloride, (7-azabenzotriazol-1-yloxy) trispyrrolidinophosphonium hexafluorophosphate (PyAOP), albumin from bovine serum (BSA), 2, 3, 5-triphenyltetrazolium chloride (TTC), porous graphitic carbon (PGC) and cellulose microcrystalline (MCC) were obtained from Sigma-Aldrich (Merck, Darmstadt). The empty cartridges and frits were obtained from Tianjin BonnaAgela Technologies Inc (Tianjin) and were utilized to self-packed PGC and MCC columns (each column was 100 mg filler) for solid phase extraction (SPE). All preparations of reaction solutions were carried out with Milli-Q ultra-pure water (Merck, Millipore).

## Mice model of middle cerebral artery occlusion (MCAO)

Male C57BL/6J mice (8–10 weeks, 22–25 g) from Cyagen Biosciences were housed under controlled conditions (12:12 reversed light cycle, 22 ± 1 °C, 50 ± 5% humidity) with ad libitum access to food/water. Transient focal cerebral ischemia was induced via 60-min middle cerebral artery occlusion (MCAO) under isoflurane anesthesia (2% induction, 1% maintenance) [13], as approved by the ethics committee of the Shenzhen Second People’s Hospital and China Technology Industry Holdings (Shenzhen) Co., LTD (202400118).

## Tissue processing and serum sample acquisition

Following successful MCAO surgery under effective anesthesia, TTC staining of coronal brain section confirmed successful modeling in MCAO mice. Based on TTC results, brain tissues from ischemic or non-ischemic areas were selected for subsequent experiments. The samples were ultrasonically lysed, then the intracellular components were collected for N-glycan pre-treatment [13].

Consecutive AIS patients presenting within 6 h of symptom onset were recruited from the Emergency Department of Shenzhen Second People’s Hospital, with diagnosis confirmed through NIHSS assessment and diffusion-weighted MRI [14, 15]. Age-/sex-matched controls were selected from routine physical examination participants. All human studies complied with Declaration of Helsinki principles under approval of the Ethics Committee of Shenzhen Second People’s Hospital (No. 20181106007) with written informed consent.

## N-glycan Preparation

The 100 µg of tissue protein or 5 µL serum were mixed with 10 µL of glycobuffer (10x) and water to 100 µL in total. Rapid deglycosylation of 20 min using microwave-assistance was performed as our previous study [16]. Then the solution diluted by 500 µL of 5% ACN containing 0.1% TFA was loaded onto a PGC SPE column preconditioned with 3 mL of ACN and equilibrated in 3 mL of 5% ACN containing 0.1% TFA. After another wash using 3 mL of 5% ACN containing 0.1% TFA, the released glycans were eluted using 1 mL of 40% ACN containing 0.1% TFA, and then the solution was collected and dried for further use.

## Methylamination and purification

The released glycans were further labeled with methylamination for a better detection sensitivity of all types of glycan structures and purified by MCC SPE as previous report [17] with some modifications. Briefly, the dried glycans were dissolved by 25 µL of DMSO solution containing 1 M methylamine hydrochloride and 0.5 M N-methylmorpholine and 25 µL of PyAOP (50 mM in DMSO) solution. Mixtures were vortexed and reacted at room temperature for 40 min. Then the solution diluted by 500 µL 1-butanol/ethanol/H_2_O (4:1:1, v/v/v) for further purification using MCC SPE.

### MALDI-TOF-MS analysis

Matrix assisted laser desorption ionization time-of-flight mass spectrometry (MALDI-TOF-MS, AB SCIEX, Concord, Canada) was performed for N-glycan analysis. 50% ACN was used to dissolved the dry samples. 1 µL of each sample was mixed with 1 µL fresh prepared Super-DHB solution (10 mg/mL in 50% ACN contain 1mM NaOH). Mixtures were then loaded onto the MALDI plate for analysis The MS spectrometer was operated in positive mode as (M + Na) ^+^ ions, the spectra were accumulated by 1200 laser shots, and the instrument was calibrated before the detection using peptide standards. MS/MS was performed in the positive ion mode for the target glycans with a fixed laser intensity of 6000 kW/cm^2^. The data of N-glycans were further processed using Data Explorer 4.0 (AB SCIEX, Concord, Canada). The raw data are publicly available via the GlycoPOST accession.

### N-glycan identification and abbreviation

N-glycan derivatives were analyzed by MALDI-TOF/MS and the proposed glycan composition was further annotated according to previously published and annotated tissue and serum N-glycome profile [17–19]. Quality control was further performed to verify the effectiveness and stability of the protocol. The interday repeatability of the analytical method was less than 10% of the relative standard deviation (RSD). For each glycan sample, three technical replicates were performed under the same conditions by MALDI-TOF/MS. All typical structural types for N-glycans including high-mannose, hybrid and complex entities were covered with quantitative reproducibility of < 5% RSD. The m/z values were applied to generate target glycan structures and visualized using the DrawGlycan-SNFG (http://www.virtualglycome.org/DrawGlycan/.) [20].

## Analysis of N-glycan profiles

The abundance (S/N) of all N-glycans was normalized based on total ion current of detected GPs to determine the relative abundance of N-glycans in each sample. Then, all peaks in the generated N-glycan map were quantified and compared. For N-glycan profiles from different cohorts, statistically analysis was carried out using analysis of variance (ANOVA) for three or more group or student’s T-tests (T-test) for two groups. Orthogonal or partial least squares discriminant analysis (OPLS-DA) was used to aid in the visualization of differences, and receiver operating characteristic (ROC) and the area under the curve (AUC) analysis were used to investigate the specificity. A difference of the *p* < 0.05 was considered statistically significant with a confidence interval of 95% (95 CI). Statistical analysis were performed and visualized using the IBM SPSS Statistics 29.0 and Graph Pad Prism (Version 10.6.1).

## Results

### N-Glycan profiles in mouse brain tissue

**Fig. 2:**
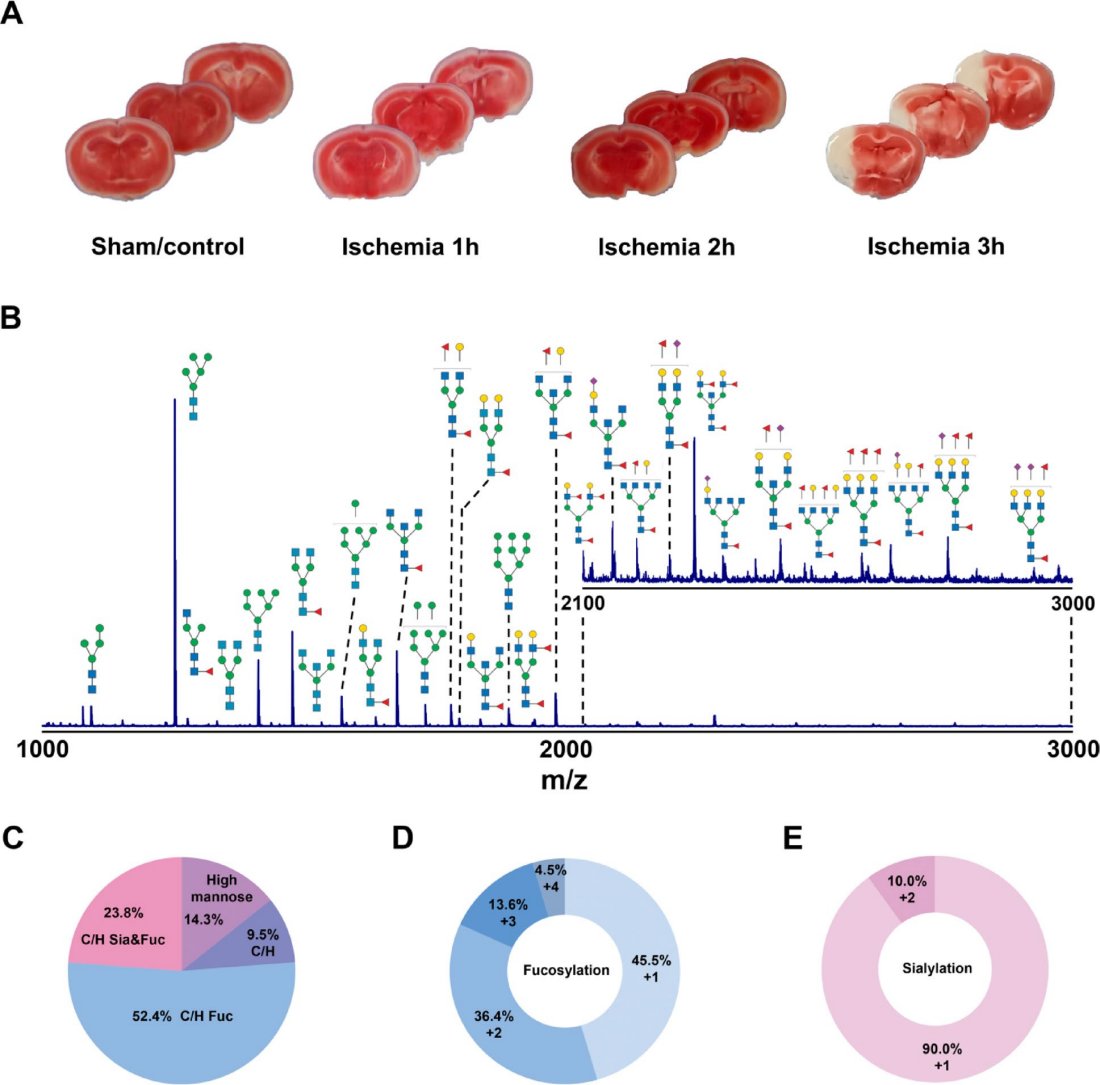
**(A)** The 2, 3, 5-triphenyltetrazolium chloride (TTC)-stained of mouse brain tissue slices from sham control and different ischemic time for 1 h, 2 h and 3 h (3 mice in each group). **(B)** The represented MALDI-MS spectrum of N-glycans in mouse brain tissue. Relative abundances of the different glycan types were detected. **(C)** Percentages of glycans belonging to each of four biosynthetic classes including high mannose (high mannose); undecorated complex/hybrid (C/H); fucosylated complex/hybrid (C/H Fuc); and fucosylated-sialylated complex/hybrid (C/H Sia and Fuc). **(D)** Percentages of fucosylated types glycans according to the degree of fucosylation (*n* = 1, 2, 3, 4). **(E)** Percentages of sialylated types glycans according to the degree of sialylation (*n* = 1, 2)

Progressive expansion of the ischemic lesion was observed with increasing occlusion duration (0–3 h), culminating in near-total hemispheric infarction at the 3-hour timepoint (Fig. 2A). This spatiotemporal progression reflects the dynamic transition from viable penumbral tissue to irreversible infarction during early AIS pathogenesis [21]. To characterize N-glycan alterations during penumbral evolution, we performed comparative glycomic analysis of: Ipsilateral (ischemic) versus contralateral (non-ischemic) hemispheres, time-resolved tissues (1 h, 2 h, and 3 h) from MCAO mice (*n* = 5 for each time point) and sham-operated control mice (*n* = 3). The 3-hour ischemic core served as spatial reference for tissue sampling. Given the functional heterogeneity of cerebral regions [19], all analyses accounted for variations between hemispheres.

Figure 2B showed the represented MALDI-MS spectrum of glycans for all mouse brain tissue, and a total of 42 distinct N-glycans (T1-T42) were detected and identified across samples (summarized in Supplementary Table S1). Glycan species were classified as the 1structural types (high-mannose, hybrid, and complex glycans), and glycosylation features (fucosylation, sialylation) [19]. All the glycan species were summarized in Supplementary Table S2. As a result, the glycome was dominated by fucosylated complex/hybrid structures (76.2%), with high-mannose (14.3%) and unmodified complex/hybrid glycans (9.5%) constituting minor fractions (Fig. 2C). Monofucosylation (45.5%) and monosialylation (90.0%) represented predominant modifications, though bifucosylated (36.4%) and bisialylated (10.0%) variants were also detected. Notably, rare multiply fucosylated species (3–4 fucose residues) were observed (Fig. 2D-E).

**Fig. 3:**
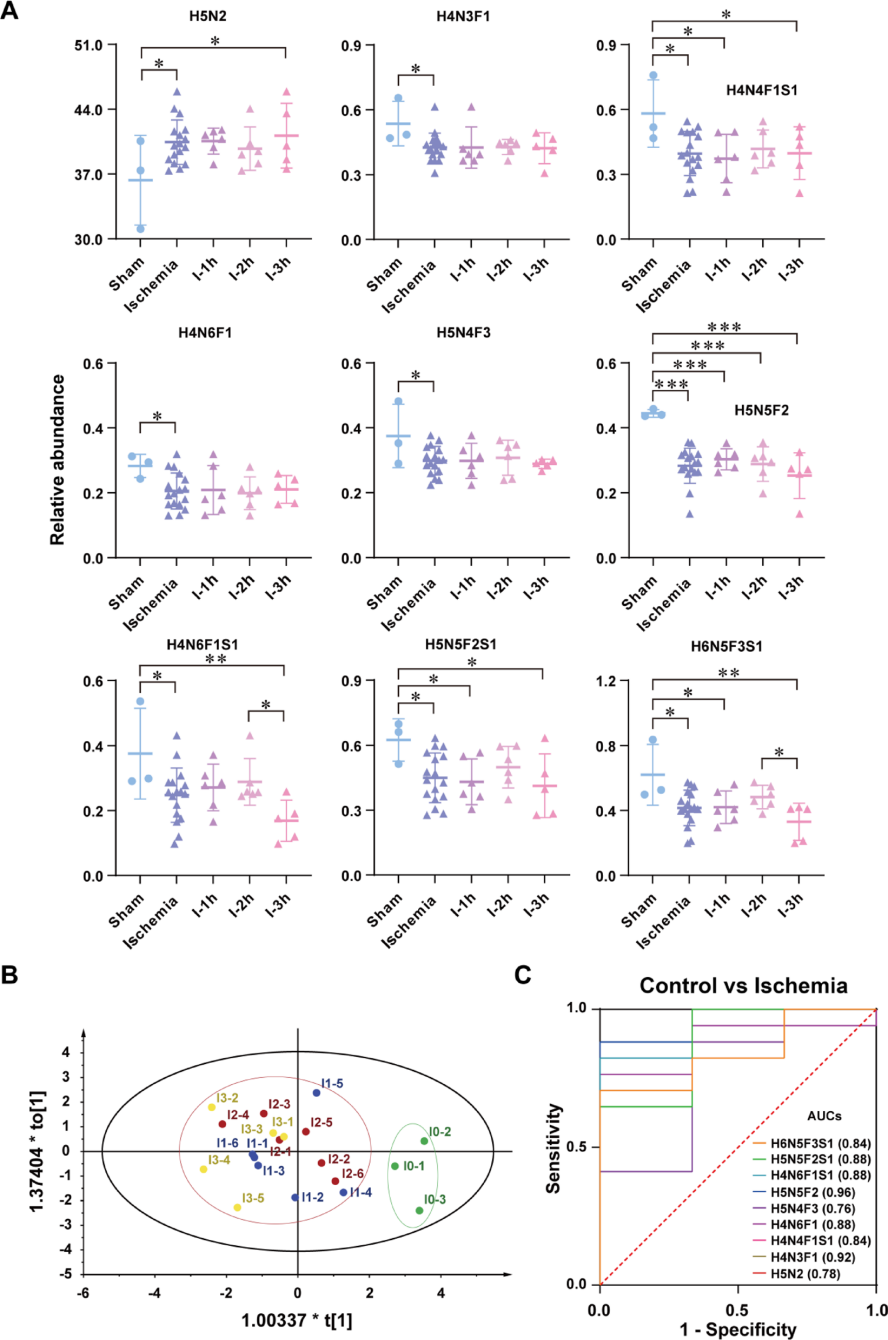
**(A**) Scatter plots of the significantly changed glycans in mouse brain tissue for control, ischemia and different ischemic time of 1 h, 2 h and 3 h, respectively. _*_, *p* < 0.05, _**_, *p* < 0.01, _***_, *p* < 0.001. **(B)** OPLS-DA of significantly changed glycans for control (labeled with I0 1–3) and different ischemic time of 1 h (labeled with I1 1–6), 2 h (labeled with I2 1–6) and 3 h (labeled with I3 1–5). **(C)** ROC analysis with AUC of significantly changed glycans for control and total ischemic samples (Control vs. Ischemia)

To compare the glycome differentiation during AIS early stage, Shapiro-Wilk test was firstly used to examine the normality for glycan profiles. It turns out that all the GPs abundance followed a normal distribution (*p* > 0.05, Supplementary Table S3). Paired T-test analysis revealed 5 N-glycans with significant interhemispheric differences (*p* < 0.05, Supplementary Table S4), likely reflecting regional glycome heterogeneity, which were excluded in following analysis. Subsequent multi-test correction (homoscedasticity, T-test, Mann-Whitney U) identified 9 ischemia-sensitive glycans (*p* < 0.05, Supplementary Tables S5-S6). ANOVA with post-hoc test using LSD analysis was further performed to evaluate their temporal changes among control, 1 h, 2 h, and 3 h ischemia (Fig. 3A, Supplementary Table S7). 8 of glycans including biantennary complex type glycans of H4N3F1, H4N4F1S1, H4N6F1, H5N4F3, H5N5F2, H4N6F1S1, H5N5F2S1 and H6N5F3S1 presented a significant down-regulation, and one oligomannose glycan of H5N2 was significantly increased. These individual glycans show significant dysregulation in ischemia vs. control, while their levels do not show a consistent, significant progressive change from 1 h to 3 h, making them poor for staging but excellent for diagnosis. For these glycans, OPLS-DA achieved clear discrimination between ischemic and normal tissues (t1 with a score of 44.4%, Fig. 3B). However, they seem to be not reliably distinguished between ischemic durations (1–3 h). ROC analysis demonstrated strong diagnostic performance (AUC = 0.76–0.96) for ischemia-specific glycans (Fig. 3C). Furthermore, results of effect size (Supplementary Table S8) showed that the Cohen’s d of all significantly changed glycan species between controls and ischemia were over 0.5, providing moderate effect for differences between two groups.

### Comparison of glycosylation features of mouse brain tissue

To delineate ischemia-induced N-glycosylation features, we systematically compared mannosylation, fucosylation, and sialylation patterns across experimental groups: sham controls, global ischemia, and temporal ischemia subgroups (I-1 h, I-2 h, I-3 h). ROC analysis was omitted for features lacking intergroup significance.

### Mannosylation

**Fig. 4:**
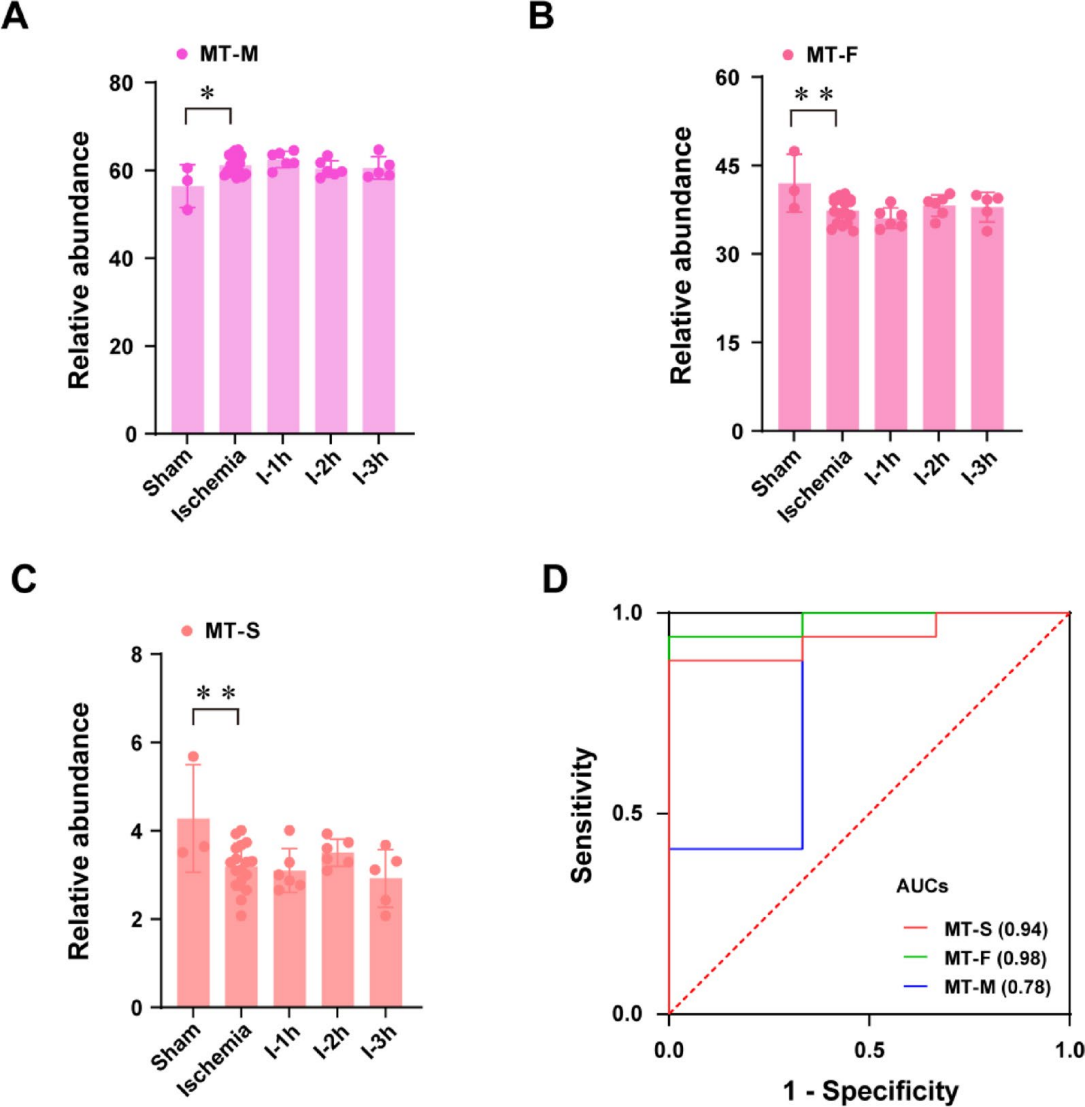
Relative abundance of the different glycan features for all glycans from mouse brain tissue (MT) among controls (sham), global ischemia (ischemia), and temporal ischemia subgroups (I-1 h, I-2 h, I-3 h). **(A)** Mannosylation (MT-M) **(B)** Fucosylation (MT-F). **(C)** Sialylation (MT-S). _*_, *p* < 0.05, _**_, *p* < 0.01. **(D)** ROC analysis with AUC of significantly changed glycan features for control and total ischemic mouse brain tissues (Control vs. Ischemia)

High-mannose glycans, essential for neurodevelopment and synaptic plasticity [22], exhibited marked ischemia sensitivity. Mannosylated species in mouse brain tissue (MT-M) showed a significant depletion in ischemic tissue (*p* < 0.05, Fig. 4A) with moderate effect (Cohen’s d of 0.542, Supplementary Table S8) and achieved diagnostic potential (AUC = 0.78, Fig. 4D). This aligns with the known role of mannose receptor (MR)-mediated glycan clearance in acute neuroinflammation.

### Fucosylation

Extensive fucosylation represents another significant structural feature of glycans. In mouse brain, fucosylated glycans are essential for proper brain development [23, 24]. As shown in Fig. 4B, ischemia-specific fucosylated glycans (MT-F) demonstrated a significant downregulation (*p* < 0.01). Notably, it exhibited a high diagnostic value (AUC = 0.98, Fig. 4D) and a high effect (Cohen’s d of 0.948, Supplementary Table S8) between controls and ischemia, potentially reflecting impaired fucose-dependent axonal guidance mechanisms.

### Sialylation

The central nervous system (CNS)-enriched Neu5Ac sialylation, essential for cell migration, neurogenesis and guidance of neurites [25], showed ischemic sensitivity. Ischemia-associated sialylation (MT-S) were markedly reduced (*p* < 0.01, Fig. 4C) with moderate effect (Cohen’s d of 0.757, Supplementary Table S8) and demonstrated exceptional diagnostic performance (AUC = 0.94, Fig. 4D), consistent with sialic acid shedding during blood-brain barrier disruption. As anticipated with extremely low expression [26, 27], Neu5Gc was undetectable, confirming murine CNS-specific sialylation patterns.

### Validation of N-glycome from human serum

Serum samples from early AIS patients including transient ischemic attack (TIA, ischemic time < 6 h) and over recanalization windows (ORW, presented outside the time window for thrombolysis/thrombectomy, 6 h < ischemic time < 12 h) and healthy controls (without ischemia) were collected and analyzed for validation. Diagnostic classifications followed the 2023 Chinese Guidelines for AIS Diagnosis and Treatment [28]. Demographic characteristics are summarized in Table 1. Variance homogeneity analysis using ANOVA with LSD test or Tamhane T2 post-hoc tests revealed no significant age or sex differences between groups (all *p* > 0.05), confirming these variables did not confound subsequent N-glycan profiling.

**Table 1:** Demographic characteristics of the ischemic stroke and healthy control participants

Characteristic	Control (C)	Transient ischemic attack (TIA, T)	Over recanalization windows (ORW, O)	*p* value (C/T)	*p* value (C/O)	*p* value (T/O)
Participants (N)	35	13	11	NA	NA	NA
Sex, (male: female)	18/17	9/4	7/4	0.279 ^a^	0.484 ^a^	0.786 ^a^
Age, (median)	60	59	66	0.741 ^b^	0.320 ^b^	0.199 ^b^

^a^ANOVA analysis with LSD test was used when the variance was equal. ^b^ANOVA analysis with Tamhane T2 test was used when the variance was unequal

**Fig. 5:**
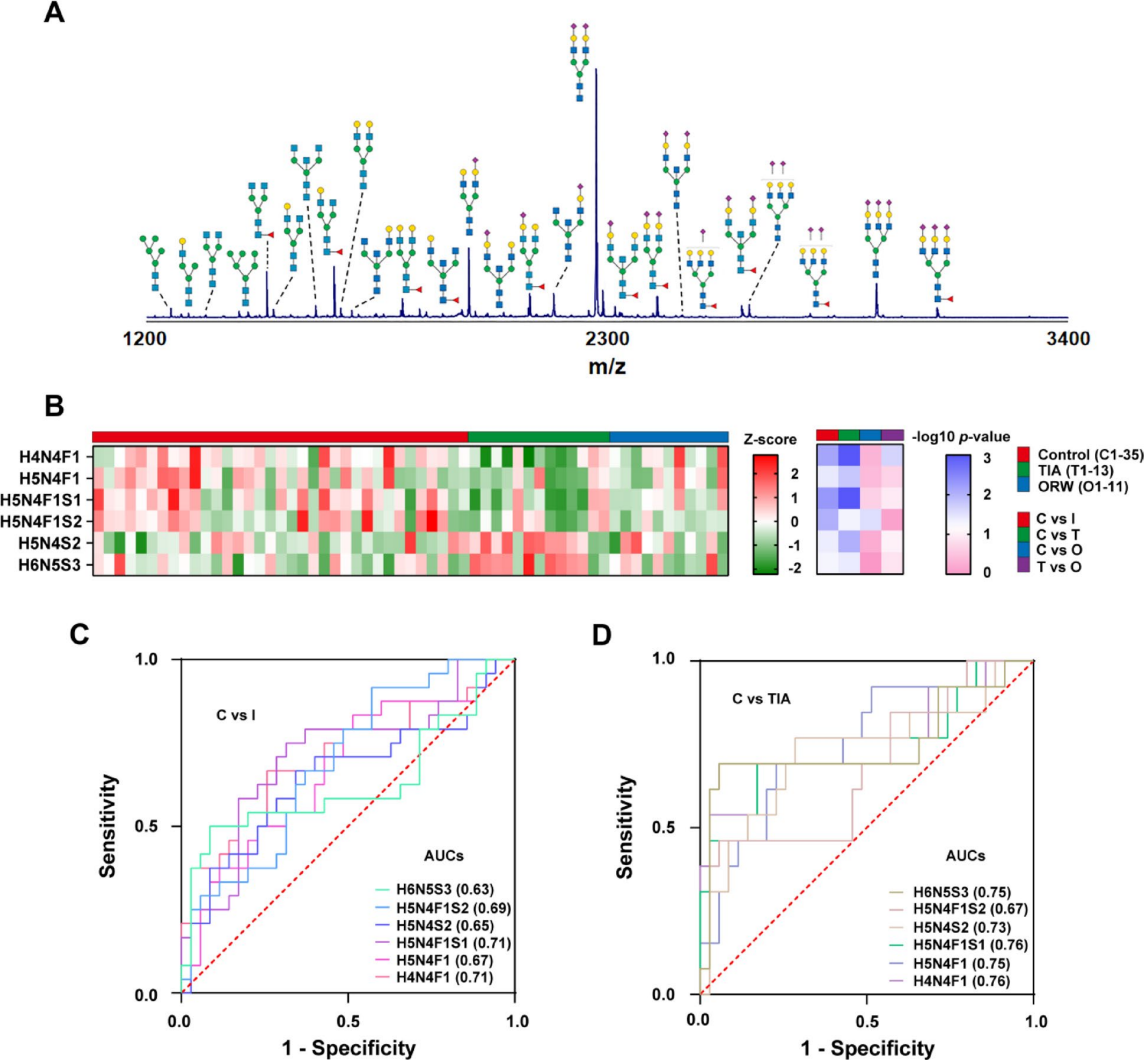
**(A)** The represented MALDI-MS spectrum of N-glycans in human serum. The composition of main glycans was depicted **(B**) Heatmap plots of the significantly changed glycans in human serum for control (C), transient ischemic attack (T), over recanalization windows (O) and total ischemic cases (I consists of T and O). ROC and AUC analysis of significantly changed glycans among the groups **(C)** C and I. **(D)** C and T

There were 31 N-glycans (S1-31) detected in all sera from TIA, ORW and control cohorts using MALDI-MS and a typical spectrum was shown in Fig. 5A. All the detected glycan ions were summarized in Supplementary Table S9. By using the same method as the mouse glycome analysis, (normality test, homoscedasticity, T-test, Mann-Whitney U, and ANOVA with post hoc test) identified six differentially expressed glycans between AIS patients and controls (*p* < 0.05, Supplementary Tables S10-S12), with low effect (Cohen’s d < 0.542, Supplementary Table S13). Heatmap visualization (Fig. 5B) demonstrated dynamic glycan alterations across disease states: fucosylated/sialylated structures H4N4F1, H5N4F1, H5N4F1S1 and H5N4F1S2 exhibited progressive decline from controls through ischemia groups, while H5N4S2 and H6N5S3 showed reciprocal increases. Similarly, consistent with the above results from mouse brain tissue, glycan levels do not show a consistent, significant progressive change from TIA to ORW cohorts as well (Supplementary Table S14). In addition, ROC analysis of candidate biomarkers revealed moderate discriminative capacity (AUC 0.63–0.76) for distinguishing early stroke states (Fig. 5C-D).

**Fig. 6:**
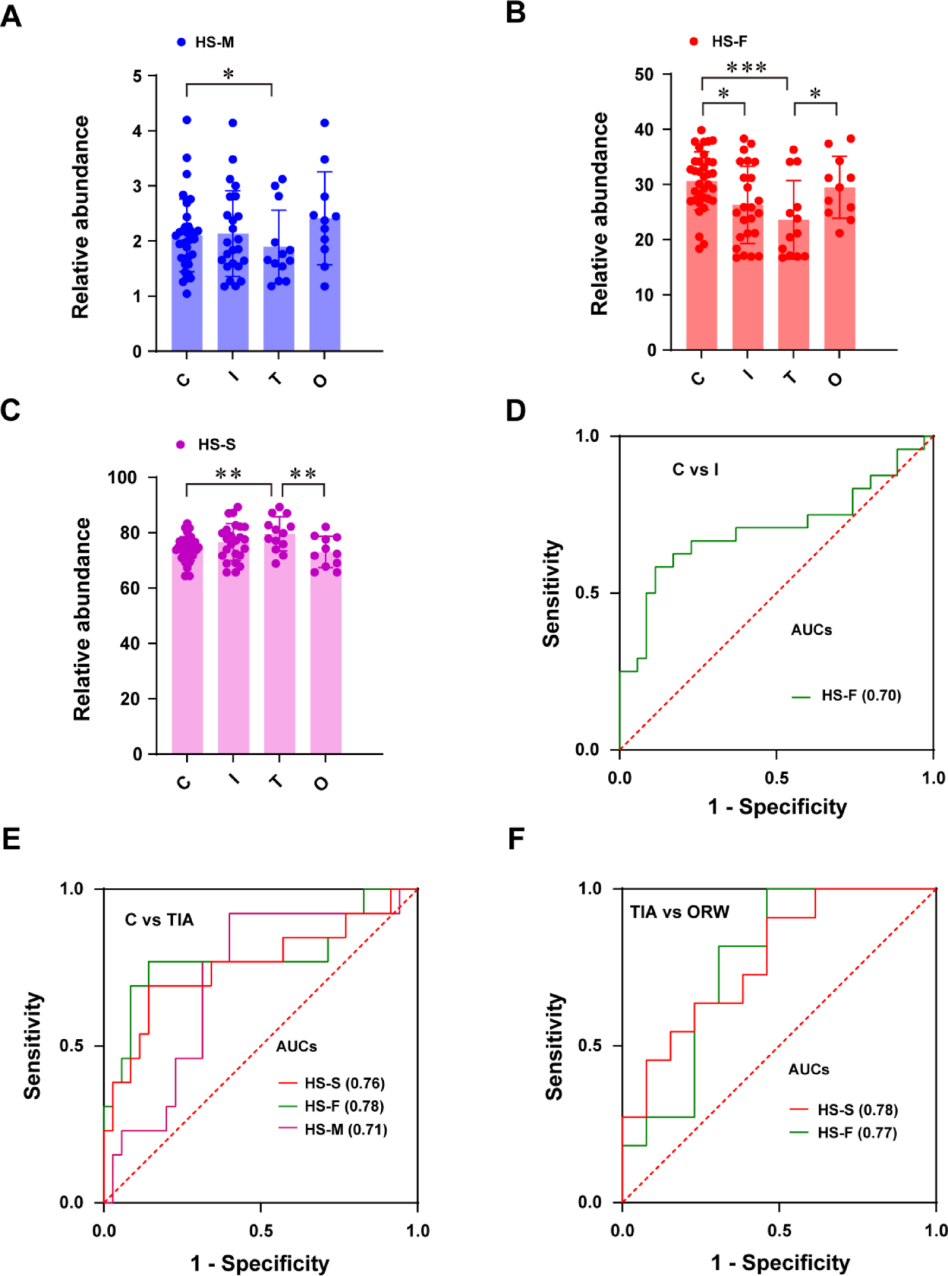
Relative abundance of different glycan features for all detected glycans from human serum (HS) among transient ischemic attack (TIA) and over recanalization windows (ORW) and healthy controls. **(A)** Mannosylation (HS-M). **(B)** Fucosylation (HS-F). **(C)** Sialylation (HS-S). _*_, *p* < 0.05, _**_, *p* < 0.01, _***_, *p* < 0.001. ROC and AUC analysis of significantly changed glycan features in serum. **(D)** Controls and early IS patients (C vs. I). **(E)** Controls and TIA patients (C vs. T). **(F)** TIA and ORW patients (T vs. O)

Further investigation of glycomic features (mannosylation [HS-M], fucosylation [HS-F], and sialylation [HS-S]) revealed significant decrease of HS-F in AIS patients versus controls (Fig. 6A-C). Comparative analysis demonstrated a diagnostic potential for HS-F (AUC 0.70–0.78 across control/ischemia, control/TIA, and TIA/ORW comparisons) and HS-S (AUC 0.74–0.76 for control/TIA and TIA/ORW), with HS-M showing more limited discriminative power (AUC 0.71 in control/TIA comparison) (Fig. 6D-F, Supplementary Table S15). ROC analysis was omitted for non-significant glycan traits among control/TIA/ORW groups. However, Cohen’s d of HS-M, HS-S, and HS-F between controls and ischemia were less than 0.5 (Supplementary Table S13), providing low effect for differences between two groups.

## Discussion

AIS remains a critical global health issue, marked by high rates of disability, morbidity, mortality, and recurrence. During the early stages of AIS, particularly in the ultra-early phase, the ischemic penumbra emerges in brain tissue that has not yet undergone complete necrosis. Timely restoration of blood flow can rescue neurons with reversible damage. However, as ischemic duration prolongs, the penumbra progressively diminishes in size [29]. Therefore, “time is brain”-earlier revascularization allows for greater preservation of the penumbra. Nevertheless, earlier intervention is associated with an increased risk of secondary injury, including oxidative stress, inflammatory responses, and even elevated cerebral hemorrhage risk [30]. Current clinical practice lacks reliable blood biomarkers capable of simultaneously evaluating revascularization efficacy and hemorrhagic transformation risk during early AIS progress. The dynamic regulation of N-glycosylation has emerged as a pivotal role in cerebral ischemic pathophysiology, modulating key processes such as neuronal apoptosis, neuroinflammation, and vascular endothelial responses following reperfusion injury [8–11]. The investigation of N-glycome during early AIS progression has the potential to fill existing knowledge gaps and enhance the accuracy of disease diagnosis and prognosis.

Through a series of rigorous statistical validation, glycome profiling revealed 9 ischemia-sensitive N-glycan species in murine brain tissue and 6 in human serum. Although structural differentiation among N-glycan was existed in mouse brain tissue, such variation does not substantially impact our conclusions, as this study primarily focuses on glycan composition rather than detailed structural isoforms. Unfortunately, significantly altered glycan structures were not found to intersect between mouse brain tissue and human serum. This discrepancy may be attributable to differences in ecological adaptation, evolutionary history, and biological functions between the two species [31–34].

In comparing ischemic glycosylation features, we observed a consistent downregulation of fucosylation across both models (human serum AUC = 0.78; murine brain tissue AUC = 0.98), implicating this modification as a conserved pathomechanistic feature in AIS. This pathway-level alteration appears more robust and physiologically relevant than the conservation of individual glycans during AIS, underscoring the value of pathway-focused over molecule-focused biomarkers. Furthermore, the conserved fucosylation signature suggests that this change arises rapidly and remains detectable within the critical 6-hour therapeutic window for intervention even though the time-from-onset for human AIS is inherently less precise than in controlled murine experiments. The reduction in fucosylation during the early phase may indicate a critical window for intervention, potentially influencing the timing of reperfusion strategies. Additionally, these changes could serve as potential early indicators for diagnostic purposes, allowing for timely identification of patients at risk for adverse outcomes following AIS. We believe this enhances the relevance of our findings in the context of clinical practice. However, the specific mechanism through which AIS induces glycosylation changes in serum proteins remain unclear.

Accumulating evidence confirms BBB disruption during AIS, facilitating the translocation of brain-derived glycoproteins into the peripheral circulation [10]. Our observations of decreased fucosylated glycans may thus reflect leakage of central nervous system components into peripheral blood. A recent nested case-control study has identified a significant association between the reduction of fucosylation and galactosylation of IgG and the development of AIS [35]. This investigation revealed that individuals with increased fucosylation exhibited a decreased risk of stroke, which suggests potential anti-inflammatory protective effects. Similarly, a 2021 study conducted on the Han Chinese population demonstrated that patients with dementia (secondary to stroke), exhibited significantly reduced levels of core fucosylation of IgG, accompanied by heightened pro-inflammatory activity [36]. Additionally, a study conducted among the hypertensive population revealed that a reduction in serum levels of bigalactose core α−1,6-fucosylated biantennary N-glycan was an independent predictor of asymptomatic cerebral infarction [37], further supporting the close association between altered fucosylation patterns and cerebrovascular injury.

In addressing the paradoxical relationship between the down-regulation of fucosylation and the inflammatory state in ischemic stroke, current scientific literature suggests that this ostensibly contradictory observation actually underscores the intricate regulatory mechanisms of fucosylation across diverse biological contexts, cell types, and pathological stages. While systemic inflammation is typically associated with increased fucosylation levels, such as through the promotion of leukocyte recruitment via the induction of selectin ligands, the specific microenvironment of ischemic stroke may exhibit down-regulation of local fucosylation. This down-regulation could be attributed to metabolic disturbances, endothelial damage, or alterations in immune cell function, thereby influencing the spatiotemporal dynamics of the inflammatory response [10]. Future studies needs to distinguish the fucosylation changes from different cell sources such as endothelial cells, immune cells, neurons, different types of sugar chains (core fucose vs. terminal fucose), and different time windows (acute phase vs. recovery phase) in order to comprehensively understand its true role in the inflammatory network of AIS.

Known glycan profile divergence is likely attributable to differential glycosyltransferase expression during biosynthesis [38]. Core fucosylation is catalyzed by FUT8, and its absence or down-regulation can significantly enhance the sensitivity of the central nervous system to inflammatory stimuli [39, 40]. Furthermore, recent research has substantiated that the lentiviral-mediated overexpression of FUT7 facilitates the glycosylation of CD44 fucosylation on the surface of bone marrow mesenchymal stem cells (BMSCs), resulting in the formation of H-cells (highly efficient E-selectin ligands). This modification significantly enhances the homing capability of BMSCs to ischemic brain regions, thereby mitigating reperfusion injury and reducing the release of inflammatory factors through the upregulation of PGE2, ultimately improving neurological function [41]. This study provides direct functional evidence for the role of fucosylation in modulating endothelial-immune cell interactions.

The down-regulation of fucosylation in AIS is not an isolated phenomenon; rather, it constitutes a positive feedback loop with neuroinflammatory amplification and endothelial barrier disruption. This process exhibits dynamic changes within a distinct temporal window, ranging from the acute to the subacute stage, and can be modulated through interventions involving exogenous fucose or enzyme-targeting strategies. Future research should concentrate on elucidating the expression profiles of critical nodes in the fucose metabolic pathway, such as FUT8 and FCSK, at various stages of stroke. This focus aims to facilitate the development of glucose-based therapeutic interventions that are specific to the temporal window of the condition.

The present study has suffered several methodological limitations that warrant consideration. First, the structural characterization of glycans was constrained by the coverage capacity of MS/MS analysis. Second, comparative analysis across tissue types was challenged by the limited availability of brain tissue specimens from AIS patients. Additionally, the generalizability of our conclusions is tempered by the relatively limited sample size (e.g. murine *n* = 3 per group) across comparative cohorts. For such small cohorts, results should be interpreted as trends rather than definitive significance. We fully acknowledge the need for precise elucidation of structure-activity relationships between specific glycan epitopes and their protein targets, as well as larger multicenter validation to refine prognostic tools integrating N-glycomics with clinical parameters. As core fucosyltransferase (FUT8) exclusively catalyzes core fucosylation of N-glycans [38], future studies may employ gene editing techniques or pharmacological interventions targeting FUT8 to investigate its upstream and downstream regulatory mechanisms in neuroinflammation following of early ischemic stroke. Furthermore, the studies could elucidate the potential mechanisms underlying the temporal correlation between glycan structure–activity relationship mapping and core fucosylation patterns in modulating inflammatory cascade in early stage of AIS. We will continue to collect serum from patients with early AIS to validate the model as an independent validation set to further improve the diagnostic model.

## Conclusion

Our study pioneers the N-glycomic comparison of early AIS progression through integrated analyses of murine brain tissue and human serum samples. The superior diagnostic performance of composite glycomic traits over individual glycans establishes these structures as potential dual-purpose indicators for clinical detection and therapeutic targeting in early stage of AIS. Key findings highlight fucosylation deficiency as a potential pathophysiological hallmark of early AIS, with a possible mechanistic link to disrupted glycan-mediated inflammatory regulation and impaired cellular stress response pathways. This work not only provides a methodological framework for translational glycomics but also offers potential mechanistic insights that bridge molecular glycosylation changes to clinical AIS pathology.

## Supplementary Information

Supplementary Material 1

## Data Availability

The raw MS glycomics data generated in this study have been deposited in the GlycoPOST database [42] under accession code https://glycopost.glycosmos.org/entry/GPST000577.
