# Supplementary material for: N-glycomics profiling reveals alteration of fucosylation in early acute ischemic stroke from mouse brain tissue to human serum
Source: Clin Proteomics. 2026 Jan 28;23:10. doi: 10.1186/s12014-025-09578-w (PMC12924593; doi:10.1186/s12014-025-09578-w)
Supplement: Supplementary file 1 — Supplementary Material 1 [file 12014_2025_9578_MOESM1_ESM.doc]

**Supplementary material**

**N-glycomics profiling reveals alteration of fucosylation in early acute ischemic stroke from mouse brain tissue to human serum**

Yike Wu,^1†^ Linghui Hu,^2†^ Jianlin Huang,^1†^ Yunxue Zhong,^2^ Kangcheng Li,^1^ Zhou Qiu,^2^ Li Su,^3*^ Yuan Zhang,^2*^ Wenlan Liu^2^, ^4 *^

^1^ Department of Laboratory Medicine, Shenzhen Institute of Translational Medicine, The First Affiliated Hospital of Shenzhen University, Shenzhen Second People's Hospital, Shenzhen, 518035, China

^2^ Department of Neurosurgery, The First Affiliated Hospital of Shenzhen University, Shenzhen Second People's Hospital, Shenzhen, 518035, China

^3^ Department of Neurosurgery, South China Hospital Affiliated to Shenzhen University, Shenzhen, 518055, China

^4^ Medical Genetics Center，Shenzhen Maternity & Child Healthcare Hospital, Shenzhen 518028, China

†These authors contributed equally to this work

*Corresponding authors:

Dr. Wenlan Liu, Department of Neurosurgery, The First Affiliated Hospital of Shenzhen University, Shenzhen Second People's Hospital, Shenzhen, China. Email: wlliu@szu.edu.cn.

Dr. Yuan Zhang, Department of Neurosurgery, ShenZhen Key Laboratory of Neurosurgery, Shenzhen Second People's Hospital/the First Affiliated Hospital of Shenzhen University; Shenzhen, China. Email: zhangyuan2019@email.szu.edu.cn.

Dr. Li Su, Department of Neurosurgery, South China Hospital Affiliated to Shenzhen University, Shenzhen University, Shenzhen, China. Email: suli297@szu.edu.cn

**List of Supplementary Material**

**Table S1.** Detected N-glycan ions from mouse tissues by MALDI-MS. Glycan ions was annotated according to previously published and annotated tissue N-glycome profile.

**Table S2.** Glycan species classification according to structural types

**Table S3.** Normal distribution for N-glycome from tissues of mouse brain

**Table S4.** Paired T-test for detected N-glycan ions from ischemic and non-ischemic tissues of mouse brain

**Table S5**. Homogeneity of variance for N-glycans from control and ischemic tissues of mouse brain

**Table S6.** Significantly changed N-glycans from mouse tissue of control and ischemic samples

**Table S7.** Significantly changed N-glycans from mouse brain tissue between control and ischemic groups

**Table S8.** Effect sizes of significantly changed N-glycans and glycosylation features from mouse brain tissue between control and ischemic groups

**Table S9.** Detected N-glycan ions from human sera by MALDI-MS.Glycan ions was annotated according to previously published and annotated serum N-glycome profile.

**Table S10.** Normal distribution for N-glycome from serum of human

**Table S11.** Homogeneity of variance for N-glycans from control and ischemic serum of human

**Table S12.** Significantly changed N-glycans from human sera of control and ischemic samples

**Table S13.** Effect sizes of significantly changed N-glycans and glycosylation features from human serum between control and ischemic groups

**Table S14.** Significantly changed N-glycans from human serum between control and different ischemic stage groups

**Table S15.** Glycosylation types of detected glycans from human serum of control and ischemic samples

**Table S1.** Detected N-glycan ions from mouse tissues by MALDI-MS. Glycan ions was annotated according to previously published and annotated tissue N-glycome profile.

| **N-glycan ion (T)** | ***m/z* (M+Na^+^)** | **Chemical composition** | **Proposed Depiction** |
| --- | --- | --- | --- |
| T-1 | 1095.3698 | H4N2 | 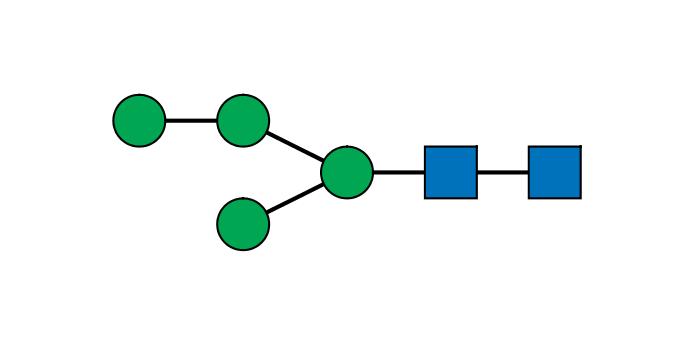 |
| T-2 | 1257.4226 | H5N2 | 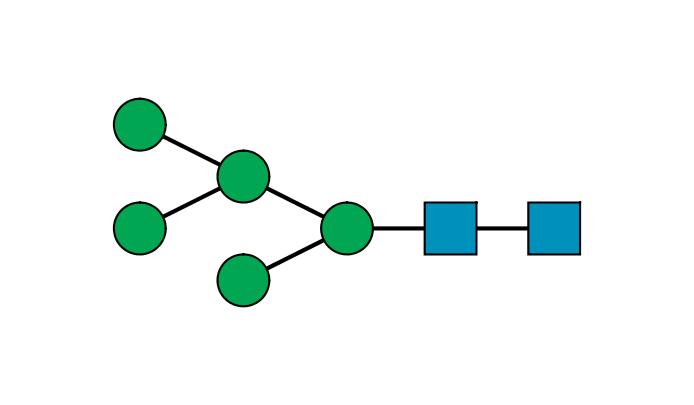 |
| T-3 | 1282.4543 | H3N3F1 | 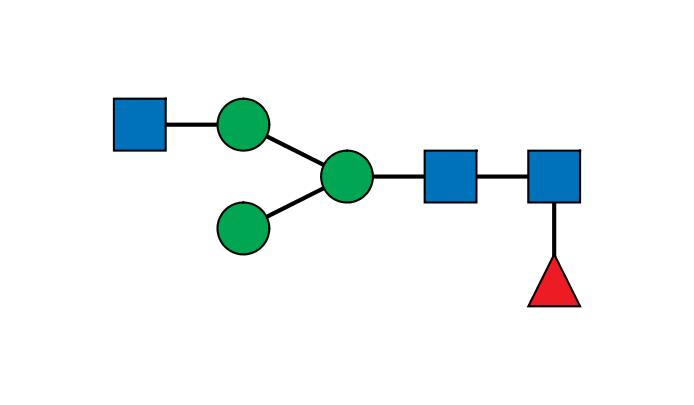 |
| T-4 | 1339.4757 | H3N4 | 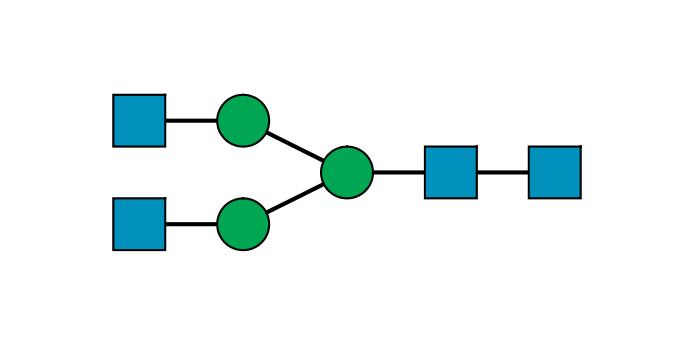 |
| T-5 | 1419.4755 | H6N2 | 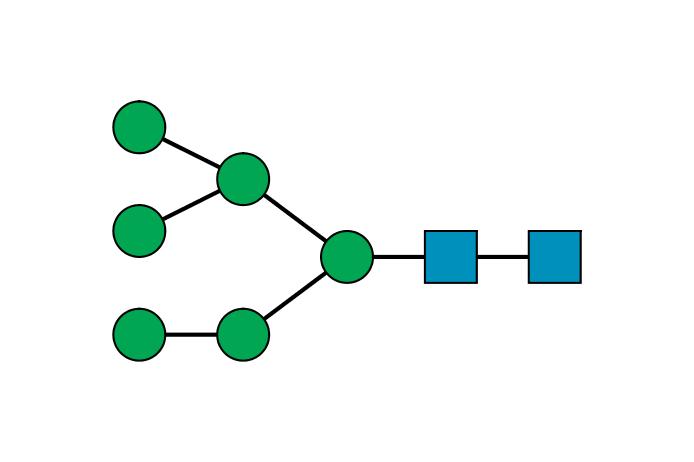 |
| T-6 | 1444.5071 | H4N3F1 | 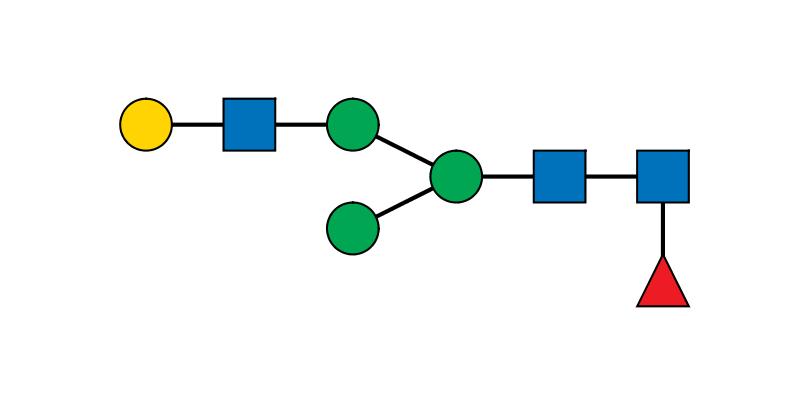 |
| T-7 | 1460.5020 | H5N3 | 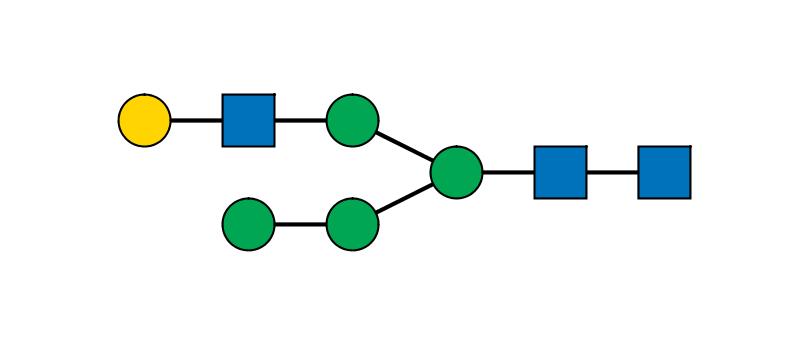 |
| T-8 | 1485.5337 | H3N4F1 | 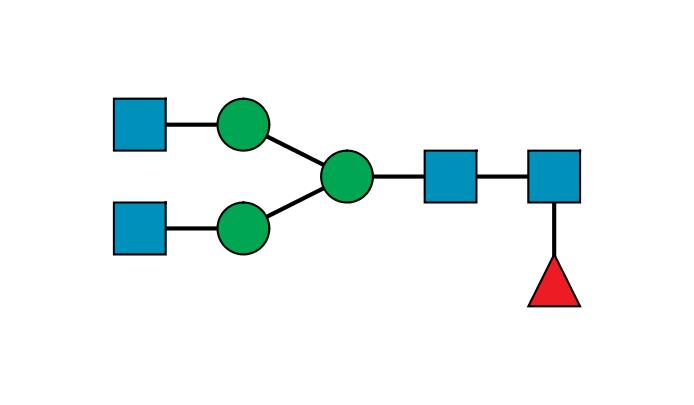 |
| T-9 | 1542.5551 | H3N5 | 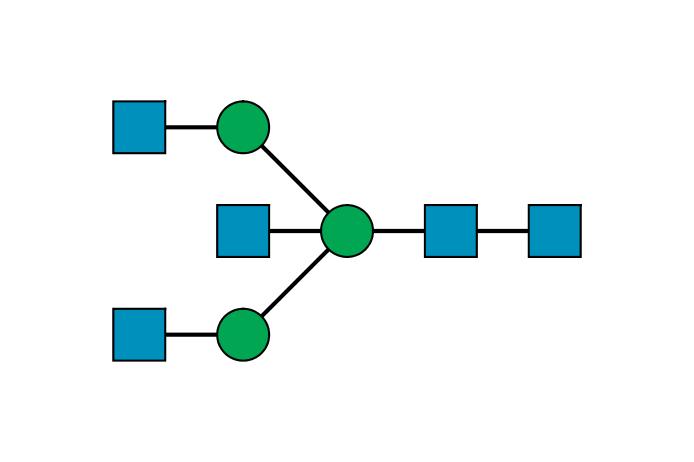 |
| T-10 | 1581.5283 | H7N2 | 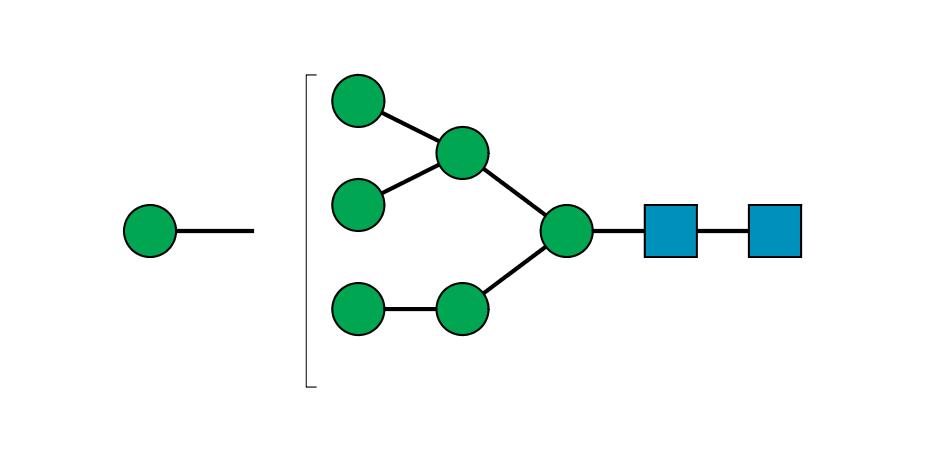 |
| T-11 | 1590.5550 | H4N3F2 | 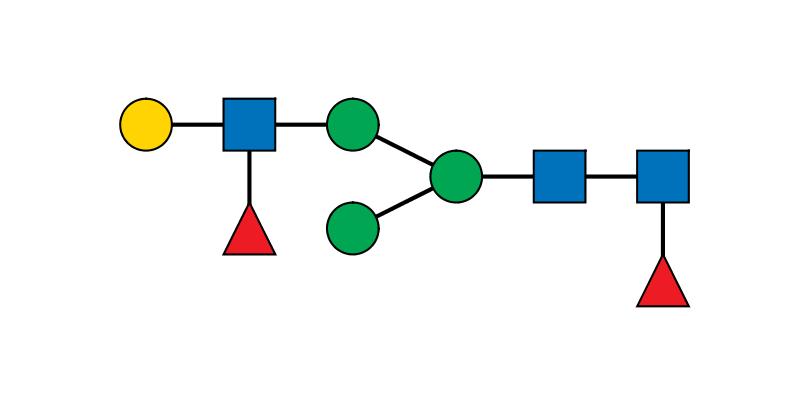 |
| T-12 | 1606.5599 | H5N3F1 | 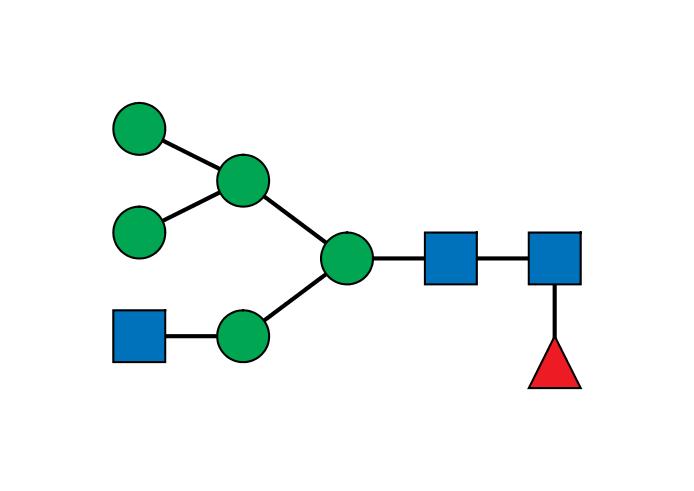 |
| T-13 | 1647.5865 | H4N4F1 | 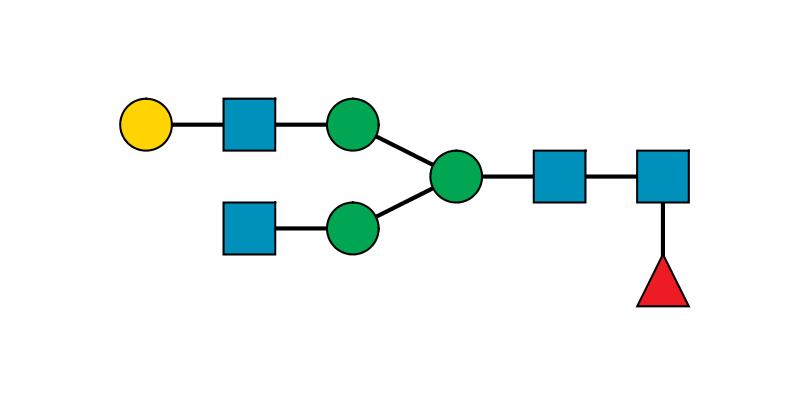 |
| T-14 | 1663.5814 | H5N4 | 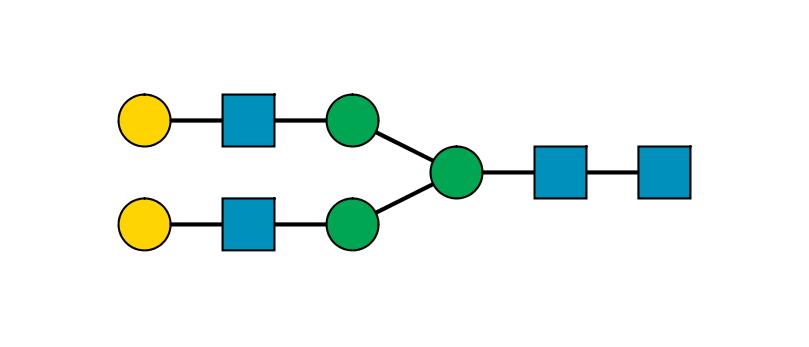 |
| T-15 | 1688.6130 | H3N5F1 | 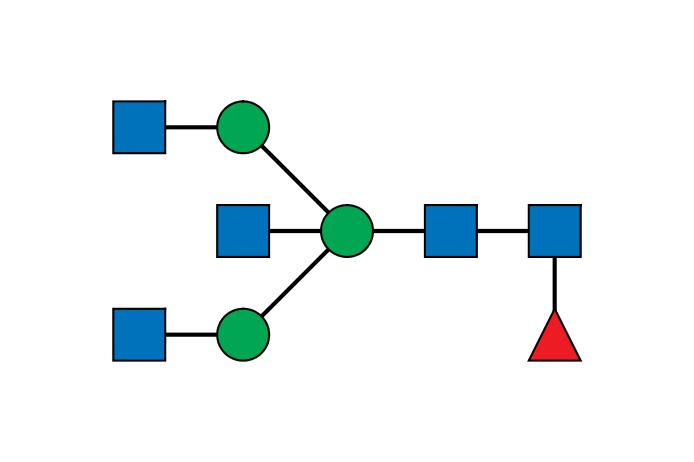 |
| T-16 | 1743.5811 | H8N2 | 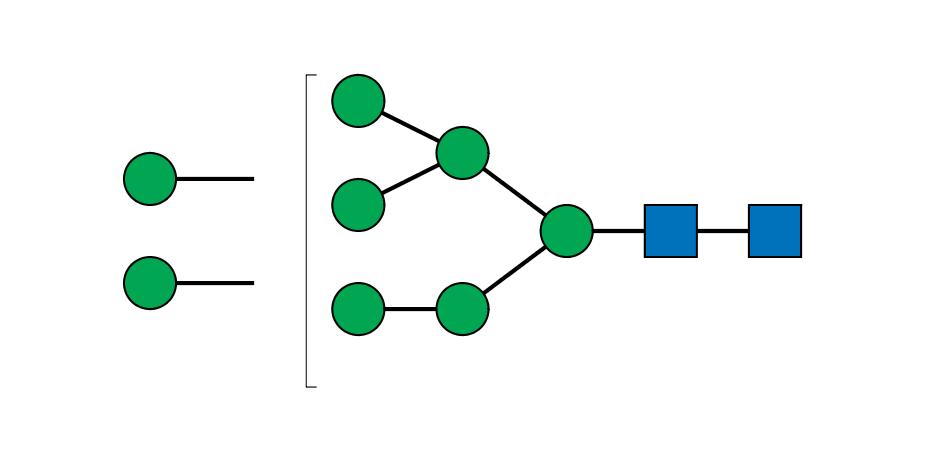 |
| T-17 | 1752.6178 | H5N3F2 | 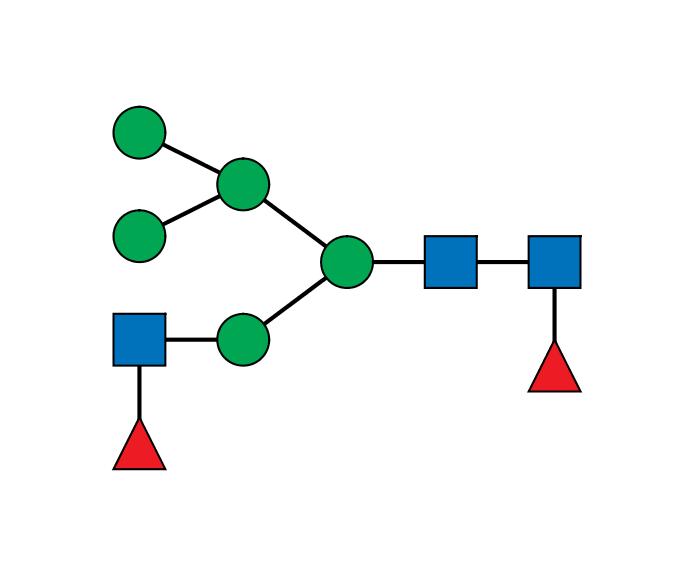 |
| T-18 | 1793.6444 | H4N4F2 | 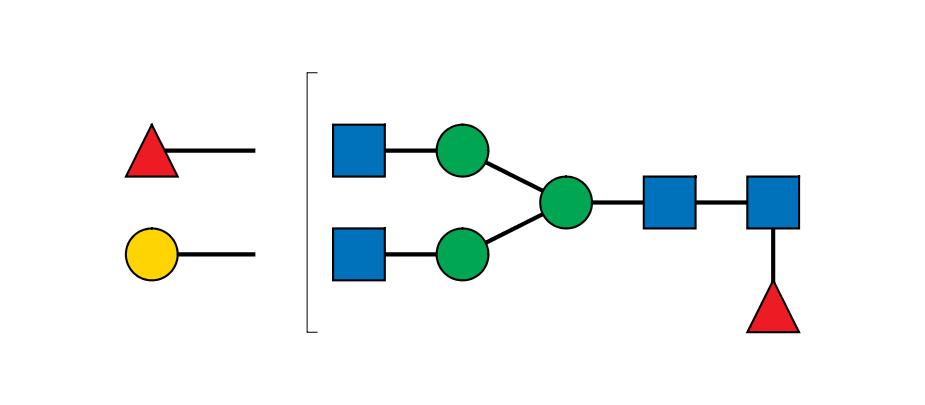 |
| T-19 | 1809.6393 | H5N4F1 | 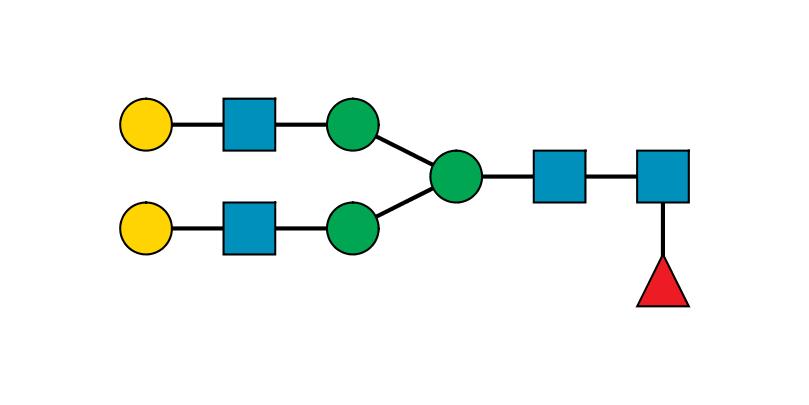 |
| T-20 | 1850.6659 | H4N5F1 | 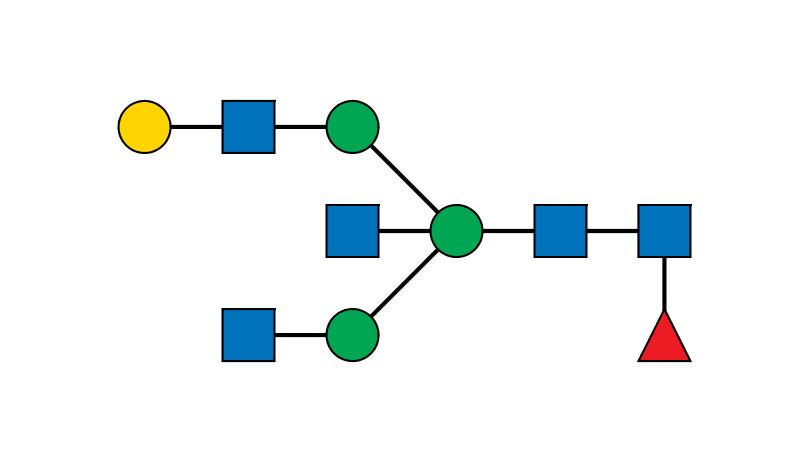 |
| T-21 | 1891.6924 | H3N6F1 | 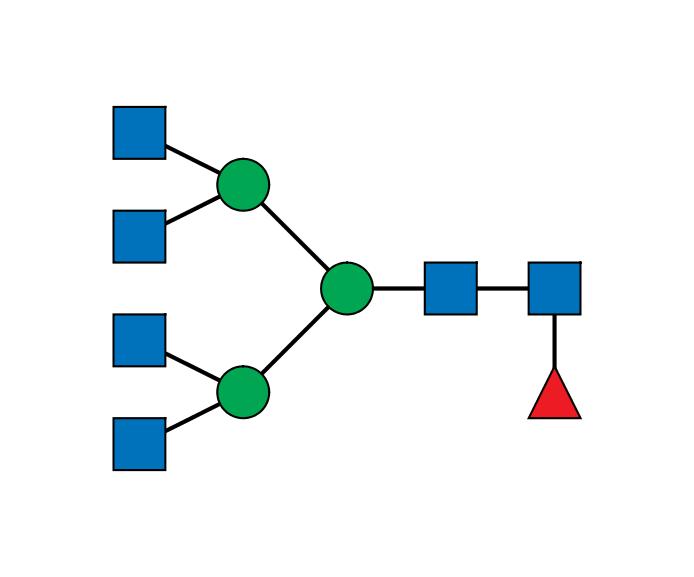 |
| T-22 | 1905.6339 | H9N2 | 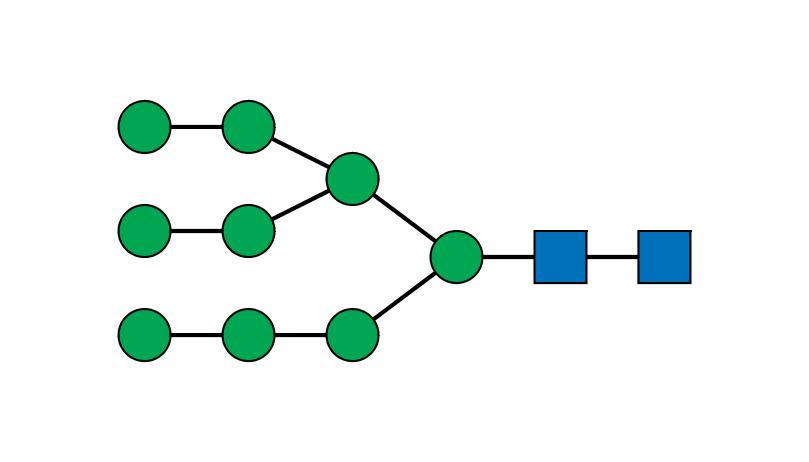 |
| T-23 | 1910.6553 | H5N3F1S1 | 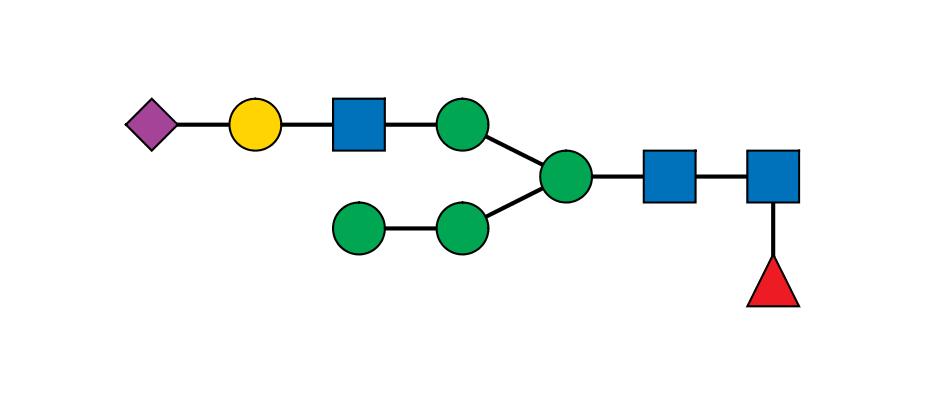 |
| T-24 | 1951.6819 | H4N4F1S1 | 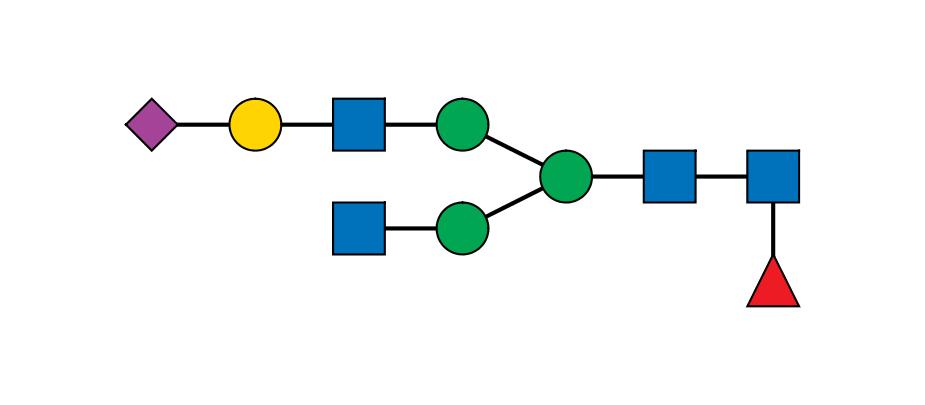 |
| T-25 | 1955.6972 | H5N4F2 | 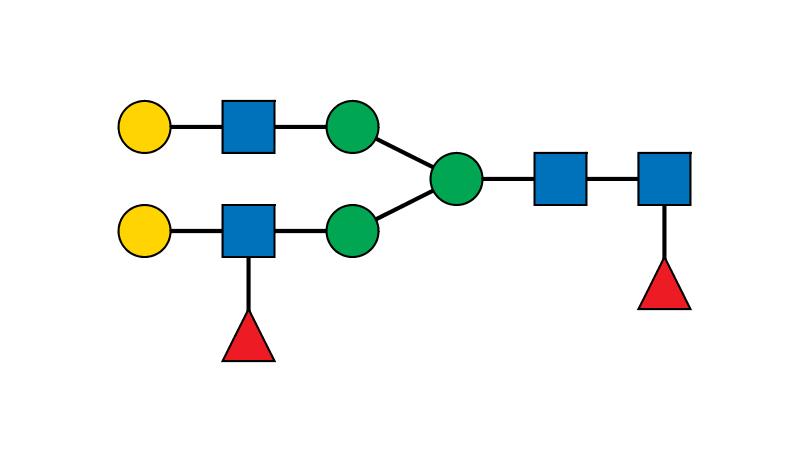 |
| T-26 | 1996.7238 | H4N5F2 | 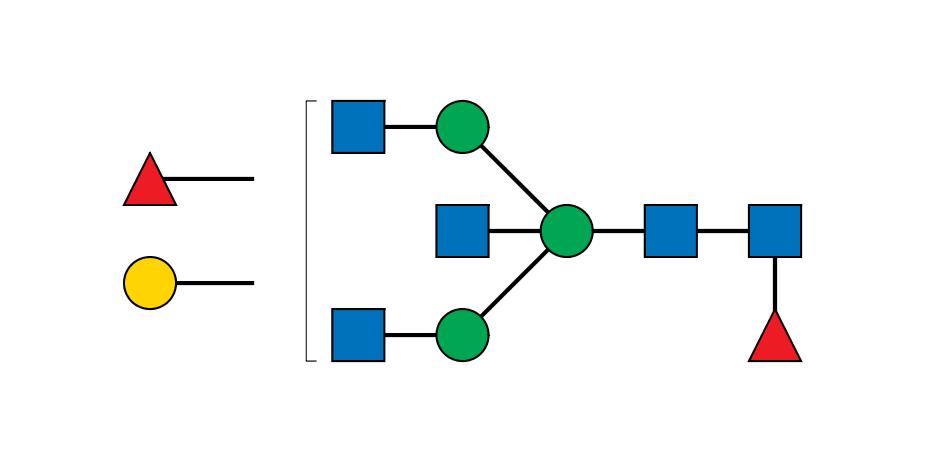 |
| T-27 | 2053.7452 | H4N6F1 | 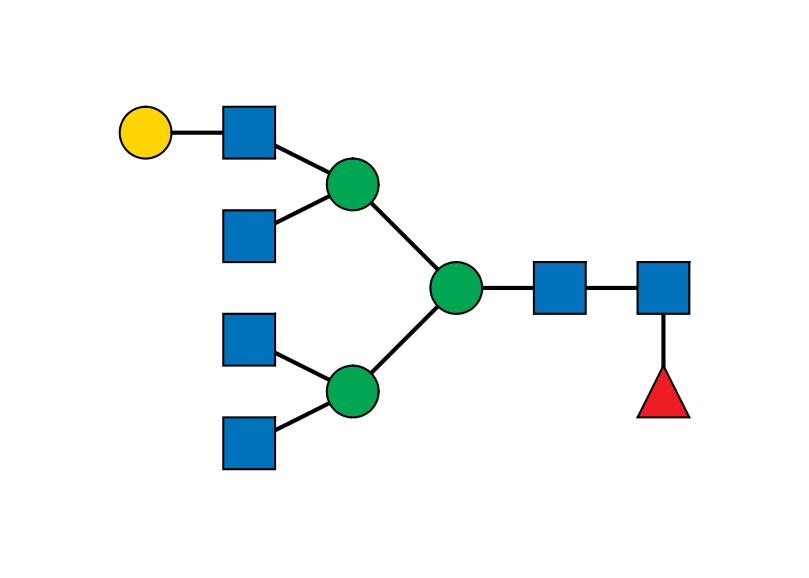 |
| T-28 | 2101.7551 | H5N4F3 | 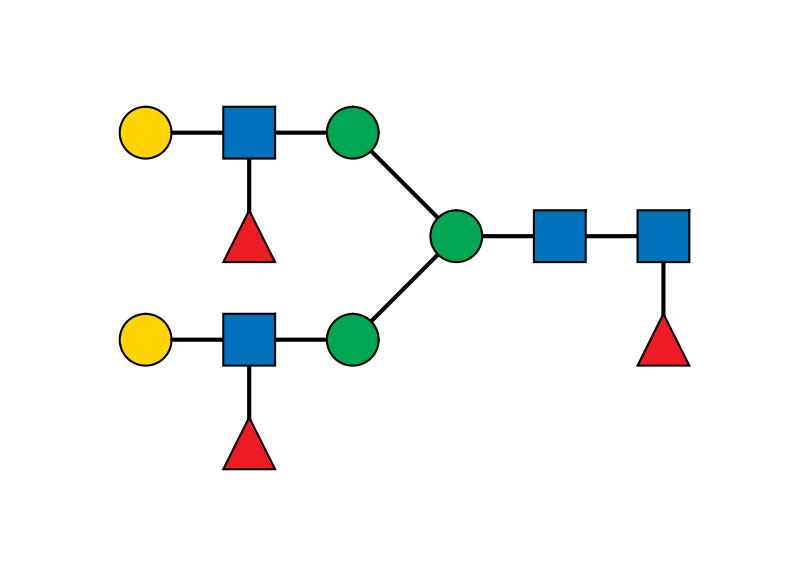 |
| T-29 | 2113.7347 | H5N4F1S1 | 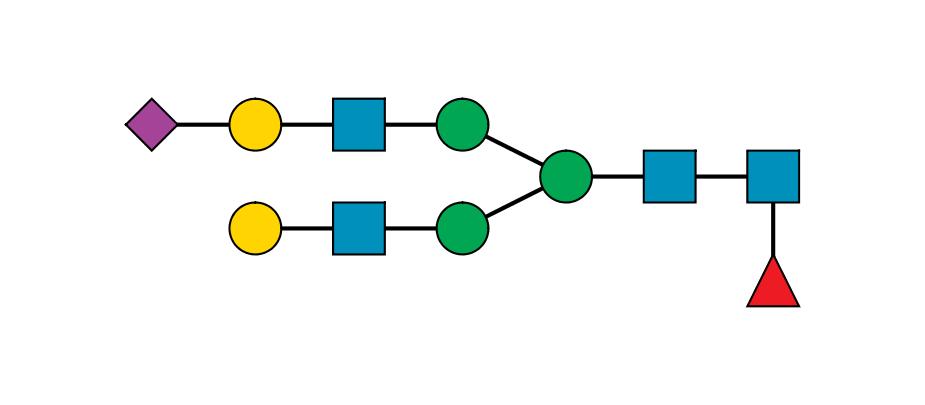 |
| T-30 | 2154.7613 | H4N5F1S1 | 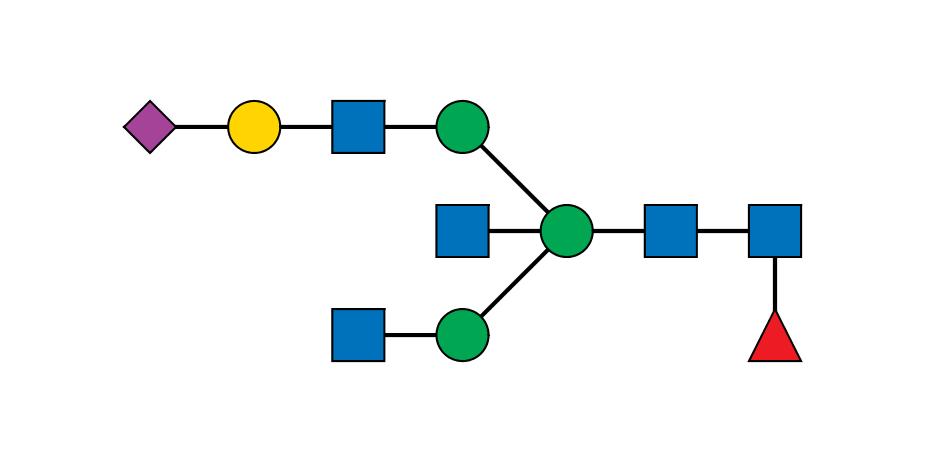 |
| T-31 | 2158.7766 | H5N5F2 | 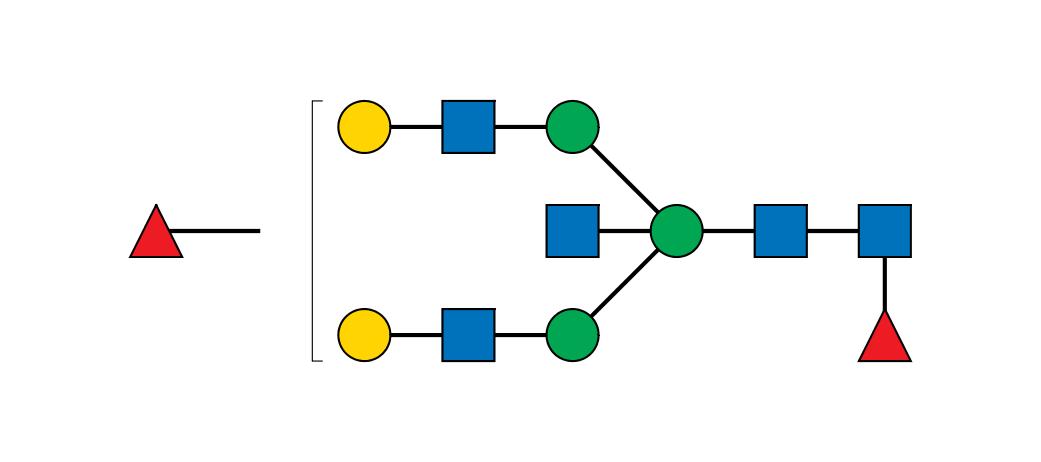 |
| T-32 | 2199.8031 | H4N6F2 | 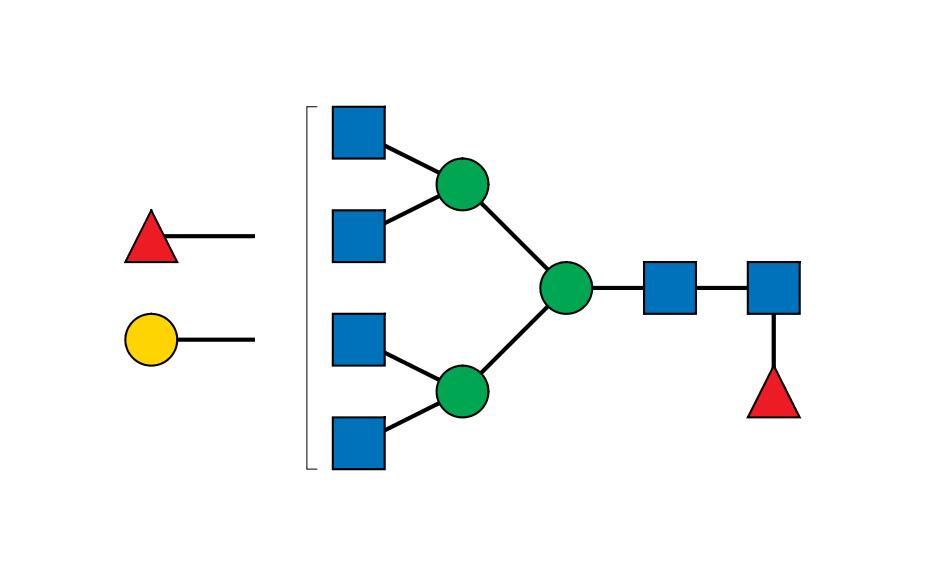 |
| T-33 | 2259.7926 | H5N4F2S1 | 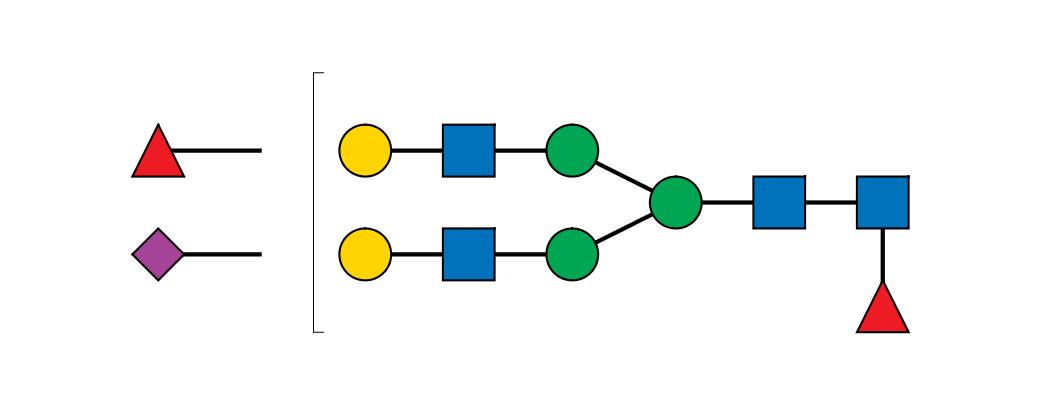 |
| T-34 | 2304.8345 | H5N5F3 | 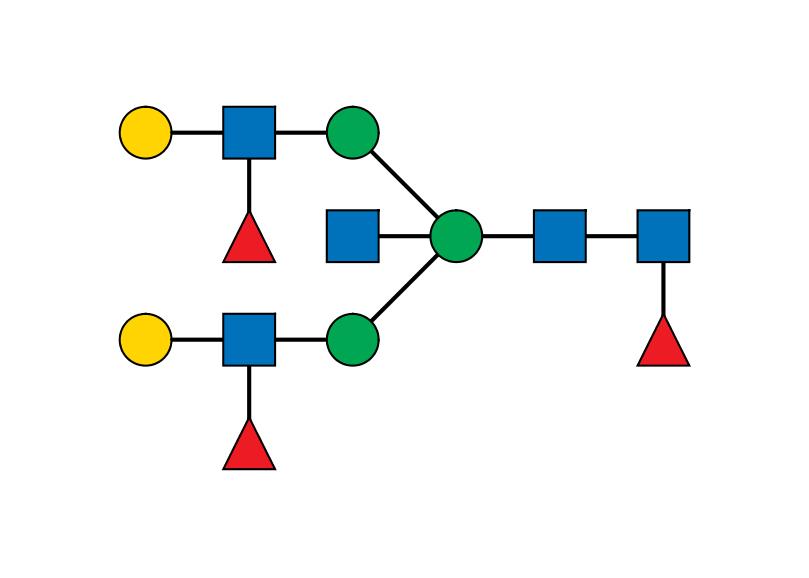 |
| T-35 | 2357.8406 | H4N6F1S1 | 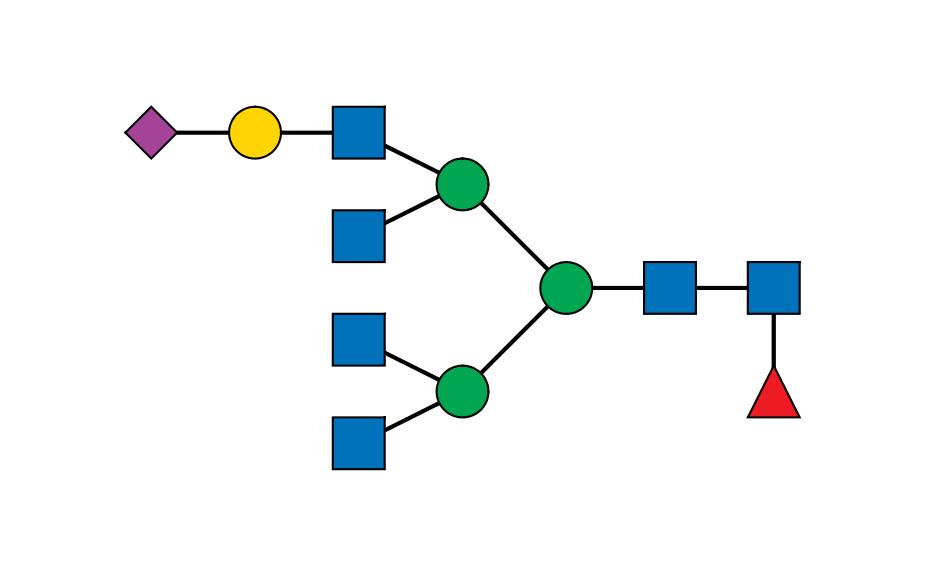 |
| T-36 | 2361.8560 | H5N6F2 | 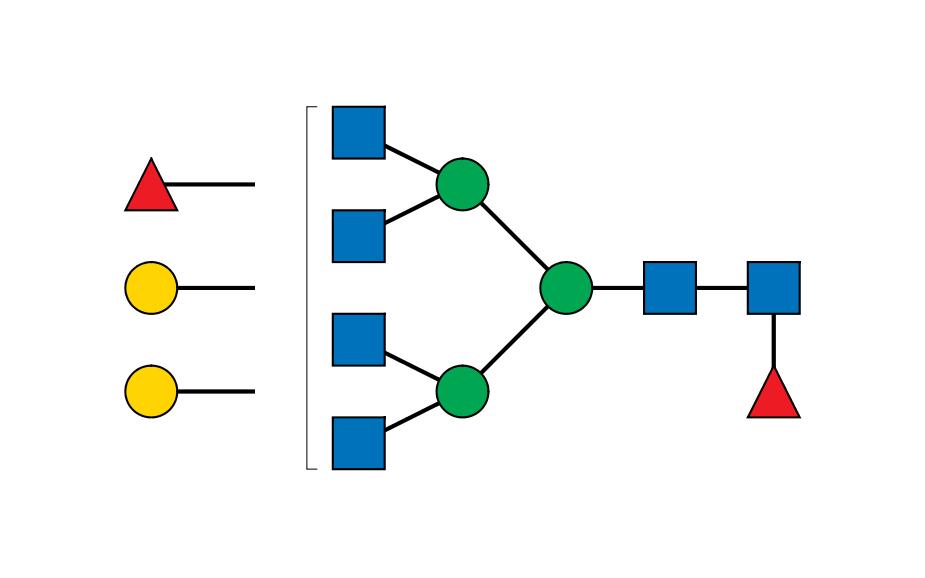 |
| T-37 | 2462.8720 | H5N5F2S1 | 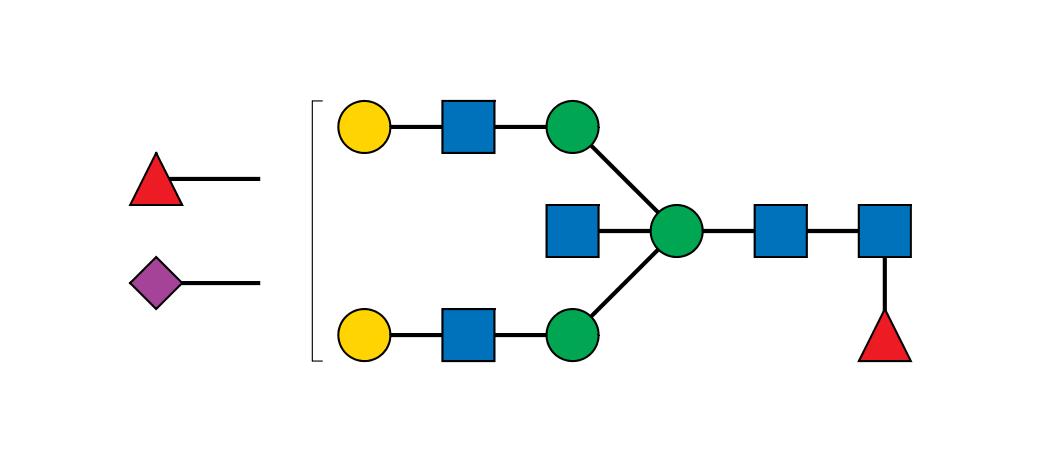 |
| T-38 | 2507.9139 | H5N6F3 | 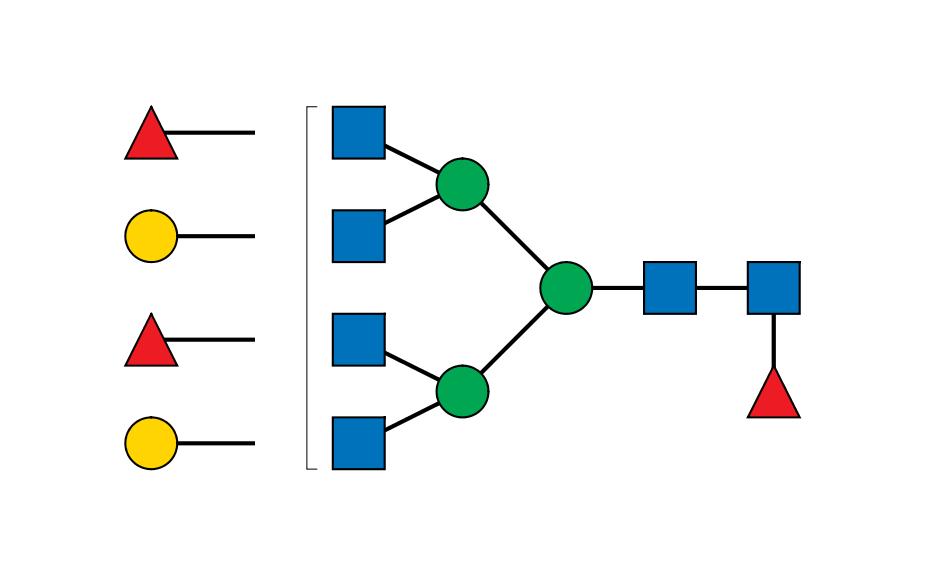 |
| T-39 | 2612.9452 | H6N5F4 | 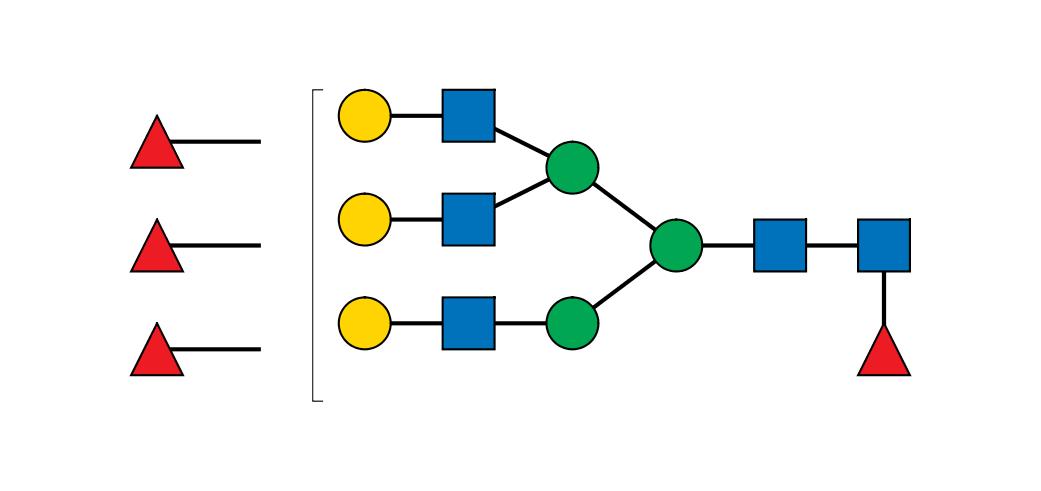 |
| T-40 | 2665.9514 | H5N6F2S1 | 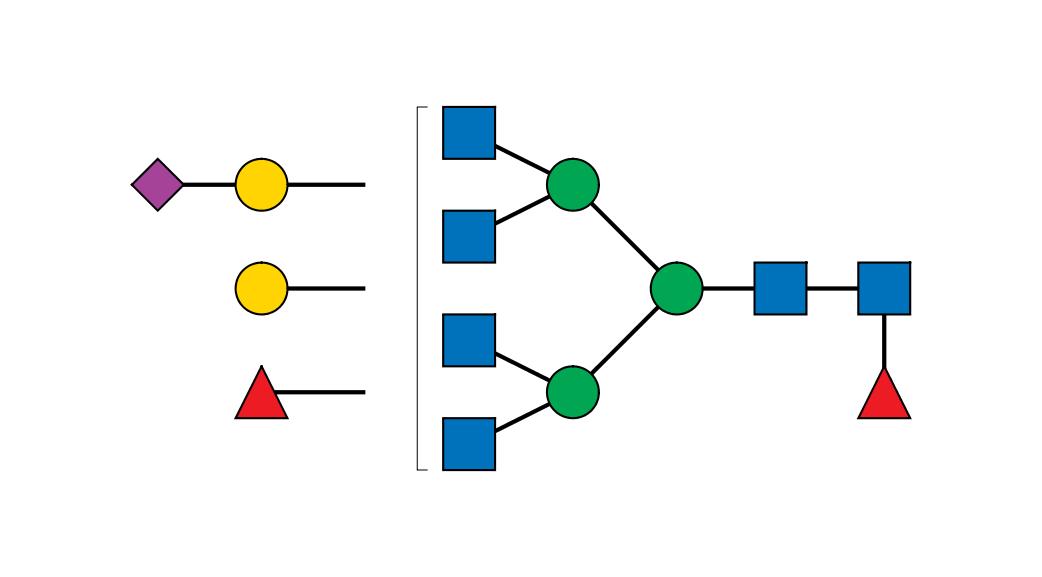 |
| T-41 | 2770.9827 | H6N5F3S1 | 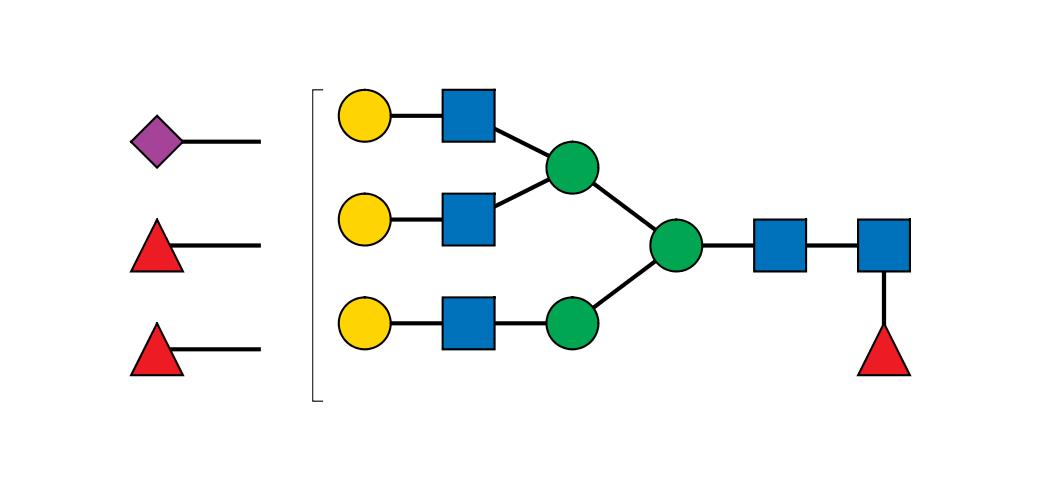 |
| T-42 | 2929.0202 | H6N5F2S2 | 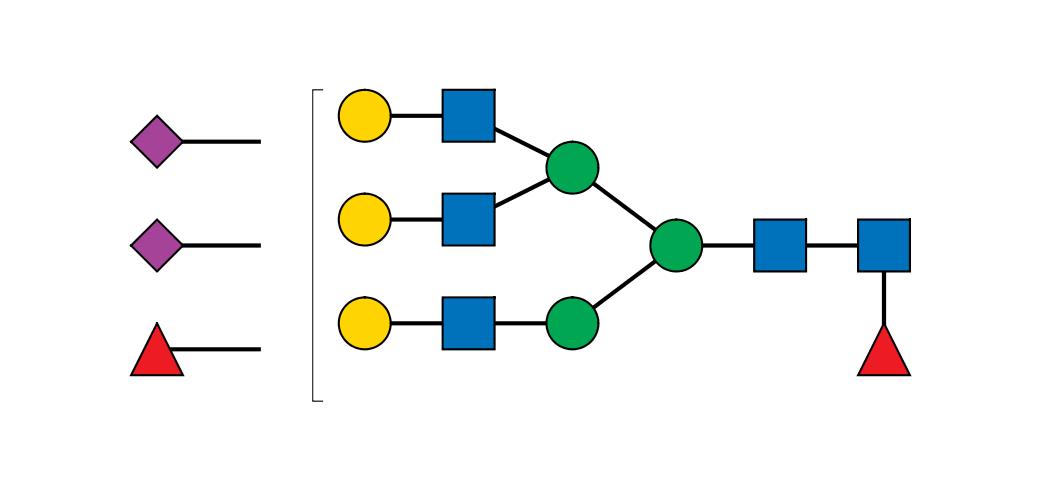 |

*Abbreviations and symbols used for N-glycans composition were as follows: Hexose (H) includes mannose (green circle) and galactose (yellow circle), N-acetylhexosamine (N) includes N-acetylglucosamine (blue square) and N-acetylgalactosamine (yellow square), N-acetylneuraminic acid (S, pink diamond) and fucose (F, red triangle). The different types of glycogroups associated with individual N-glycan compositions are distinguished numerically. Oxford annotation was used for glycan structure proposition.*

**Table S2. Glycan species classification according to structural types**

| **Structural feature** | **Glycan ions** |
| --- | --- |
| HM (High-mannose glycans) | T-1, 2, 5, 10, 16, 22 |
| C/H (undecorated complex/hybrid) | T-4, 7, 9, 14 |
| C/H Fuc (fucosylated complex/hybrid) | T-3, 6, 8, 12, 13, 15, 19, 20, 21, 27, 11, 17, 18, 25, 26, 31, 32, 36, 28, 34, 38, 39 |
| C/H Sia and Fuc (fucosylated-sialylated complex/hybrid) | T-23, 24, 29, 30, 33, 35, 37, 40, 41, 42 |
| F1 (Monofucosylated glycans) | T-3, 6, 8, 12, 13, 15, 19, 20, 21, 27 |
| F2 (Difucosylated glycans) | T-11, 17, 18, 25, 26, 31, 32, 36 |
| F3 (Trigalactosylated glycans) | T-28, 34, 38 |
| F4 (Tetragalactosylated glycans) | T-39 |
| S1 (Monosialylated glycans) | T-23, 24, 29, 30, 33, 35, 37, 40, 41 |
| S2 (Disialylated glycans) | T-42 |

**Table S3.** Normal distribution for N-glycome from tissues of mouse brain

| **N-glycan ions (T)** | **Chemical composition** | **Group** | ***p* value (I vs N) ^a^** |
| --- | --- | --- | --- |
| T-1 | H4N2 | Control | 0.351 |
|  |  | Ischemia | 0.833 |
| T-2 | H5N2 | Control | 0.635 |
|  |  | Ischemia | 0.319 |
| T-3 | H3N3F1 | Control | 0.821 |
|  |  | Ischemia | 0.338 |
| T-4 | H3N4 | Control | 0.962 |
|  |  | Ischemia | 0.857 |
| T-5 | H6N2 | Control | 0.835 |
|  |  | Ischemia | 0.979 |
| T-6 | H4N3F1 | Control | 0.148 |
|  |  | Ischemia | 0.112 |
| T-7 | H5N3 | Control | 0.438 |
|  |  | Ischemia | 0.554 |
| T-8 | H3N4F1 | Control | 0.840 |
|  |  | Ischemia | 0.101 |
| T-9 | H3N5 | Control | 0.391 |
|  |  | Ischemia | 0.732 |
| T-10 | H7N2 | Control | 0.387 |
|  |  | Ischemia | 0.920 |
| T-11 | H4N3F2 | Control | 0.372 |
|  |  | Ischemia | 0.986 |
| T-12 | H5N3F1 | Control | 0.060 |
|  |  | Ischemia | 0.397 |
| T-13 | H4N4F1 | Control | 0.482 |
|  |  | Ischemia | 0.196 |
| T-14 | H5N4 | Control | 0.984 |
|  |  | Ischemia | 0.258 |
| T-15 | H3N5F1 | Control | 0.374 |
|  |  | Ischemia | 0.060 |
| T-16 | H8N2 | Control | 0.151 |
|  |  | Ischemia | 0.075 |
| T-17 | H5N3F2 | Control | 0.231 |
|  |  | Ischemia | 0.574 |
| T-18 | H4N4F2 | Control | 0.954 |
|  |  | Ischemia | 0.927 |
| T-19 | H5N4F1 | Control | 0.446 |
|  |  | Ischemia | 0.525 |
| T-20 | H4N5F1 | Control | 0.308 |
|  |  | Ischemia | 0.442 |
| T-21 | H3N6F1 | Control | 0.558 |
|  |  | Ischemia | 0.545 |
| T-22 | H9N2 | Control | 0.792 |
|  |  | Ischemia | 0.625 |
| T-23 | H5N3F1S1 | Control | 0.485 |
|  |  | Ischemia | 0.529 |
| T-24 | H4N4F1S1 | Control | 0.626 |
|  |  | Ischemia | 0.959 |
| T-25 | H5N4F2 | Control | 0.974 |
|  |  | Ischemia | 0.380 |
| T-26 | H4N5F2 | Control | 0.730 |
|  |  | Ischemia | 0.104 |
| T-27 | H4N6F1 | Control | 0.328 |
|  |  | Ischemia | 0.085 |
| T-28 | H5N4F3 | Control | 0.567 |
|  |  | Ischemia | 0.757 |
| T-29 | H5N4F1S1 | Control | 0.754 |
|  |  | Ischemia | 0.265 |
| T-30 | H4N5F1S1 | Control | 0.095 |
|  |  | Ischemia | 0.757 |
| T-31 | H5N5F2 | Control | 0.680 |
|  |  | Ischemia | 0.160 |
| T-32 | H4N6F2 | Control | 0.363 |
|  |  | Ischemia | 0.435 |
| T-33 | H5N4F2S1 | Control | 0.283 |
|  |  | Ischemia | 0.284 |
| T-34 | H5N5F3 | Control | 0.888 |
|  |  | Ischemia | 0.473 |
| T-35 | H4N6F1S1 | Control | 0.345 |
|  |  | Ischemia | 0.183 |
| T-36 | H5N6F2 | Control | 0.143 |
|  |  | Ischemia | 0.359 |
| T-37 | H5N5F2S1 | Control | 0.356 |
|  |  | Ischemia | 0.417 |
| T-38 | H5N6F3 | Control | 0.106 |
|  |  | Ischemia | 0.358 |
| T-39 | H6N5F4 | Control | 0.289 |
|  |  | Ischemia | 0.288 |
| T-40 | H5N6F2S1 | Control | 0.488 |
|  |  | Ischemia | 0.526 |
| T-41 | H6N5F3S1 | Control | 0.783 |
|  |  | Ischemia | 0.654 |
| T-42 | H6N5F2S2 | Control | 0.938 |
|  |  | Ischemia | 0.909 |

***^a^****Shapiro-Wilk test with* *a confidence interval of 95% was used to assess the normal distribution.*

**Table S4.** Paired T-test for detected N-glycan ions from ischemic and non-ischemic tissues of mouse brain

| **N-glycan ions (T)** | **Chemical composition** | ***p* value (I vs N) ^a^** |
| --- | --- | --- |
| T-1 | H4N2 | 0.860 |
| T-2 | H5N2 | 0.794 |
| T-3 | H3N3F1 | 0.151 |
| T-4 | H3N4 | 0.757 |
| T-5 | H6N2 | 0.825 |
| T-6 | H4N3F1 | 0.399 |
| T-7 | H5N3 | 0.290 |
| T-8 | H3N4F1 | 0.187 |
| T-9 | H3N5 | 0.058 |
| T-10 | H7N2 | 0.212 |
| T-11 | H4N3F2 | 0.008 |
| T-12 | H5N3F1 | 0.824 |
| T-13 | H4N4F1 | 0.818 |
| T-14 | H5N4 | 0.705 |
| T-15 | H3N5F1 | 0.002 |
| T-16 | H8N2 | 0.915 |
| T-17 | H5N3F2 | 0.617 |
| T-18 | H4N4F2 | 0.007 |
| T-19 | H5N4F1 | 0.690 |
| T-20 | H4N5F1 | 0.443 |
| T-21 | H3N6F1 | 0.033 |
| T-22 | H9N2 | 0.051 |
| T-23 | H5N3F1S1 | 0.358 |
| T-24 | H4N4F1S1 | 0.511 |
| T-25 | H5N4F2 | 0.250 |
| T-26 | H4N5F2 | 0.530 |
| T-27 | H4N6F1 | 1.000 |
| T-28 | H5N4F3 | 0.761 |
| T-29 | H5N4F1S1 | 0.672 |
| T-30 | H4N5F1S1 | 0.958 |
| T-31 | H5N5F2 | 0.649 |
| T-32 | H4N6F2 | 0.497 |
| T-33 | H5N4F2S1 | 0.462 |
| T-34 | H5N5F3 | 0.003 |
| T-35 | H4N6F1S1 | 0.907 |
| T-36 | H5N6F2 | 0.887 |
| T-37 | H5N5F2S1 | 0.619 |
| T-38 | H5N6F3 | 0.673 |
| T-39 | H6N5F4 | 0.889 |
| T-40 | H5N6F2S1 | 0.830 |
| T-41 | H6N5F3S1 | 0.517 |
| T-42 | H6N5F2S2 | 0.907 |

***^a^****Two-tailed T-test with a confidence interval of 95% was used to identify statistically significant differences.*

**Table S5.** Homogeneity of variance for N-glycans from control and ischemic tissues of mouse brain

| **N-glycan ions (T)** | **Chemical composition** | ***p* value ^a^** |
| --- | --- | --- |
| T-1 | H4N2 | 0.493 |
| T-2 | H5N2 | 0.103 |
| T-3 | H3N3F1 | 0.385 |
| T-4 | H3N4 | 0.413 |
| T-5 | H6N2 | 0.834 |
| T-6 | H4N3F1 | 0.266 |
| T-7 | H5N3 | 0.268 |
| T-8 | H3N4F1 | 0.299 |
| T-9 | H3N5 | 0.321 |
| T-10 | H7N2 | 0.269 |
| T-12 | H5N3F1 | 0.024 |
| T-13 | H4N4F1 | 0.893 |
| T-14 | H5N4 | 0.120 |
| T-16 | H8N2 | 0.042 |
| T-17 | H5N3F2 | 0.415 |
| T-19 | H5N4F1 | 0.708 |
| T-20 | H4N5F1 | 0.059 |
| T-22 | H9N2 | 0.255 |
| T-23 | H5N3F1S1 | 0.065 |
| T-24 | H4N4F1S1 | 0.322 |
| T-25 | H5N4F2 | 0.097 |
| T-26 | H4N5F2 | 0.061 |
| T-27 | H4N6F1 | 0.356 |
| T-28 | H5N4F3 | 0.053 |
| T-29 | H5N4F1S1 | 0.064 |
| T-30 | H4N5F1S1 | 0.242 |
| T-31 | H5N5F2 | 0.200 |
| T-32 | H4N6F2 | 0.294 |
| T-33 | H5N4F2S1 | 0.838 |
| T-35 | H4N6F1S1 | 0.188 |
| T-36 | H5N6F2 | 0.314 |
| T-37 | H5N5F2S1 | 0.513 |
| T-38 | H5N6F3 | 0.314 |
| T-39 | H6N5F4 | 0.342 |
| T-40 | H5N6F2S1 | 0.025 |
| T-41 | H6N5F3S1 | 0.171 |
| T-42 | H6N5F2S2 | 0.050 |

***^a^****Levene test with 95 CI was used to identify the homogeneity of variance.*

**Table S6.** Significantly changed N-glycans from mouse tissue of control and ischemic samples

| **N-glycan (T)** | **Chemical composition** | **Control (C, n=3)** | **Ischemia (I, n=17)** | ***p* value ^a^** | ***Adjusted p value ^b^*** |
| --- | --- | --- | --- | --- | --- |
| T-2 | H5N2 | 36.336 ± 4.835 | 40.437 ± 2.399 | 0.030 | 0.045 |
| T-6 | H4N3F1 | 0.535 ± 0.103 | 0.425 ± 0.067 | 0.024 | 0.054 |
| T-24 | H4N4F1S1 | 0.581 ± 0.156 | 0.395 ± 0.102 | 0.014 | 0.063 |
| T-27 | H4N6F1 | 0.283 ± 0.036 | 0.206 ± 0.056 | 0.034 | 0.038 |
| T-28 | H5N4F3 | 0.375 ± 0.098 | 0.299 ± 0.044 | 0.033 | 0.042 |
| T-31 | H5N5F2 | 0.445 ± 0.012 | 0.283 ± 0.054 | <.001 | <.001 |
| T-35 | H4N6F1S1 | 0.376 ± 0.140 | 0.247 ± 0.084 | 0.038 | 0.038 |
| T-37 | H5N5F2S1 | 0.624 ± 0.098 | 0.449 ± 0.115 | 0.024 | 0.043 |
| T-41 | H6N5F3S1 | 0.620 ± 0.188 | 0.416 ± 0.110 | 0.015 | 0.045 |

***^a^****Two-tailed T-test with 95 CI was used when the variance was equal.* ***^b^****FDR correction used Benjamini-Hochberg test.*

**Table S7.** Significantly changed N-glycans from mouse brain tissue between control and ischemic groups

| **N-glycan**  **(T)** | **Chemical**  **composition** | ***p* value**  **(C vs I1)** | ***p* value**  **(C vs I2)** | ***p* value**  **(C vs I3)** | ***p* value**  **(I1 vs I2)** | ***p* value**  **(I1 vs I3)** | ***p* value**  **(I2 vs I3)** |
| --- | --- | --- | --- | --- | --- | --- | --- |
| T-2 | H5N2 | NS | NS | .037 | NS | NS | NS |
| T-6 | H4N3F1 | NS | NS | NS | NS | NS | NS |
| T-24 | H4N4F1S1 | .020 | NS | .043 | NS | NS | NS |
| T-27 | H4N6F1 | NS | NS | NS | NS | NS | NS |
| T-28 | H5N4F3 | NS | NS | .050 | NS | NS | NS |
| T-31 | H5N5F2 | <.001 | <.001 | <.001 | NS | NS | NS |
| T-35 | H4N6F1S1 | NS | NS | .003 | NS | NS | .027 |
| T-37 | H5N5F2S1 | .029 | NS | .022 | NS | NS | NS |
| T-41 | H6N5F3S1 | .022 | NS | .003 | NS | NS | .038 |

***^a^****ANOVA analysis with LSD test was used when the variance was equal.* ***^b^****ANOVA analysis with Tamhane T2 test was used when the variance was unequal. NS, no significance* *statistically.*

**Table S8.** Effect sizes of significantly changed N-glycans and glycosylation features from mouse brain tissue between control and ischemic groups

| **N-glycan & Glycosylation** | **Chemical**  **composition** | **Confidence interval of 95 % (95 CI)** | **Cohen’s d**  **(C vs I)** |
| --- | --- | --- | --- |
| T-2 | H5N2 | 0.447 to 7.755 | 0.542 |
| T-6 | H4N3F1 | -0.205 to -0.016 | 0.564 |
| T-24 | H4N4F1S1 | -0.328 to -0.041 | 0.621 |
| T-27 | H4N6F1 | -0.147 to -0.006 | 0.528 |
| T-28 | H5N4F3 | -0.145 to -0.006 | 0.528 |
| T-31 | H5N5F2 | -0.202 to -0.060 | 0.887 |
| T-35 | H4N6F1S1 | -0.248 to -0.007 | 0.513 |
| T-37 | H5N5F2S1 | -0.323 to -0.025 | 0.564 |
| T-41 | H6N5F3S1 | -0.363 to -0.044 | 0.618 |
| MT-M | Mannosylation | 0.447 to 7.755 | 0.542 |
| MT-S | Sialylation | -1.135 to -0.251 | 0.757 |
| MT-F | Fucosylation | -1.687 to -0.549 | 0.948 |

***Table S9.*** *Detected N-glycan ions from human sera by MALDI-MS.Glycan ions was annotated according to previously published and annotated serum N-glycome profile.*

| **N-glycan ion (S)** | ***m/z* (M+Na^+^)** | **Chemical composition** | **Proposed depiction** |
| --- | --- | --- | --- |
| S-1 | 1257.4226 | H5N2 | 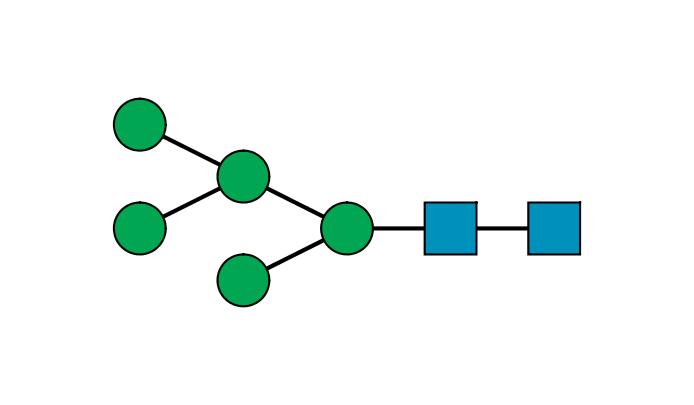 |
| S-2 | 1298.4492 | H4N3 | 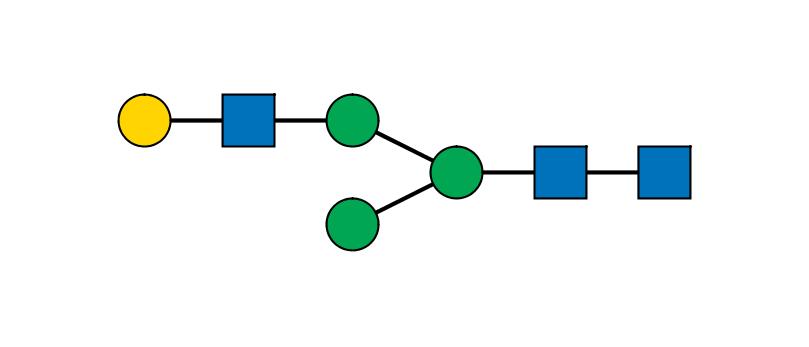 |
| S-3 | 1339.4757 | H3N4 | 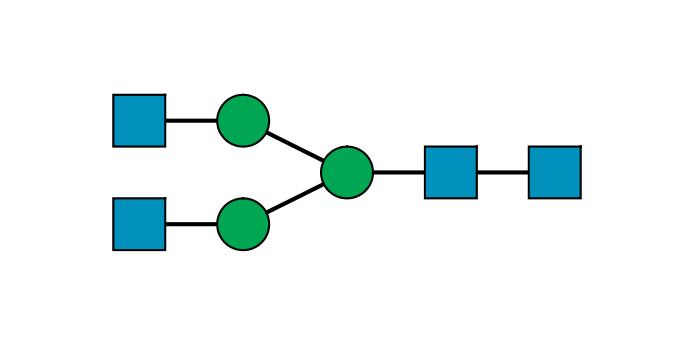 |
| S-4 | 1419.4755 | H6N2 | 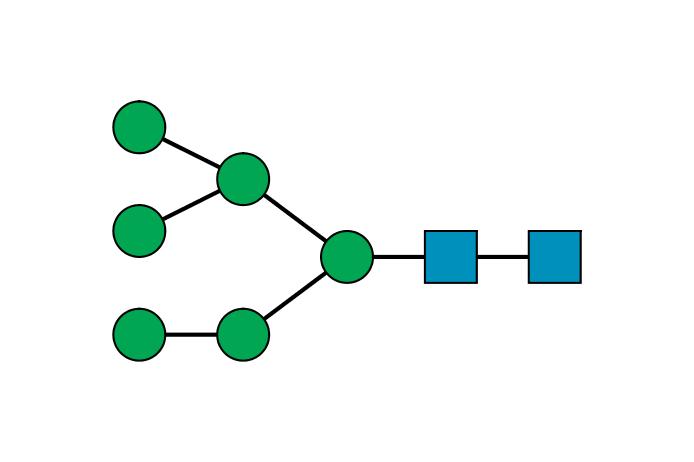 |
| S-5 | 1485.5337 | H3N4F1 | 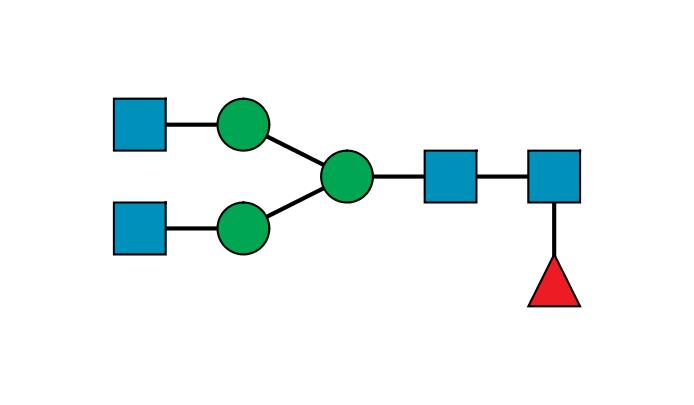 |
| S-6 | 1501.5286 | H4N4 | 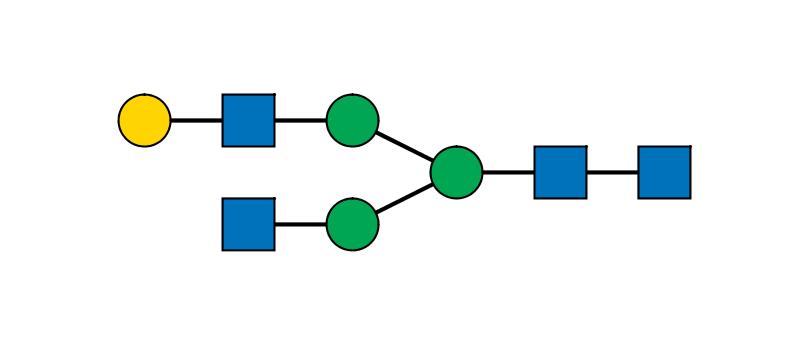 |
| S-7 | 1542.5551 | H3N5 | 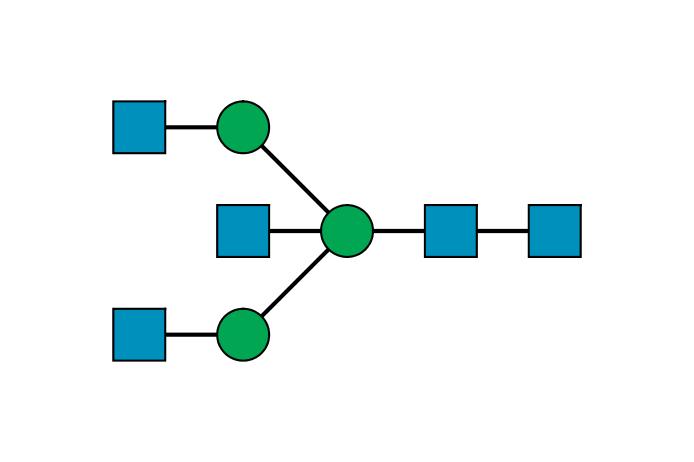 |
| S-8 | 1647.5865 | H4N4F1 | 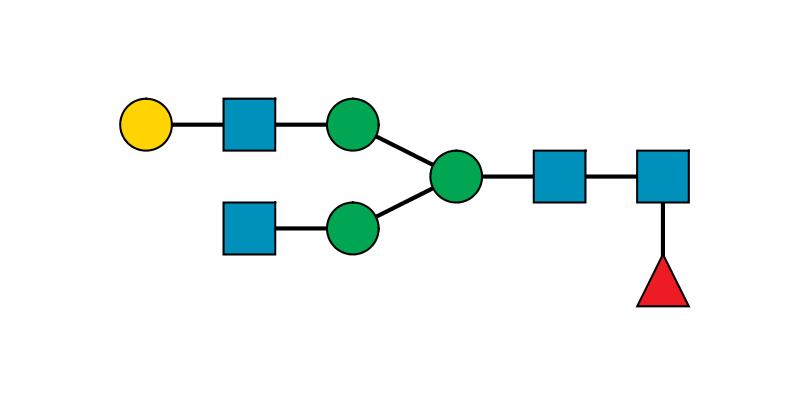 |
| S-9 | 1663.5814 | H5N4 | 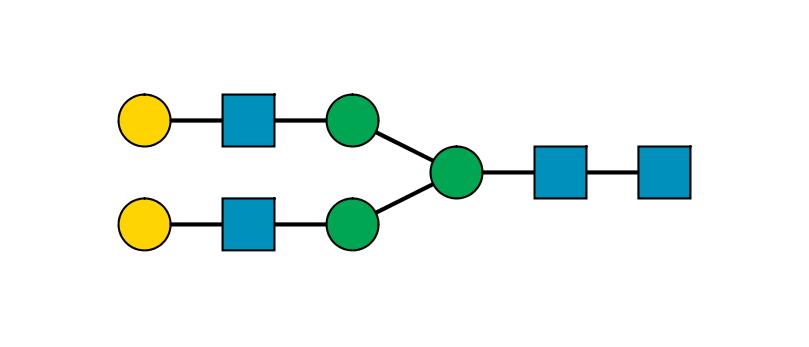 |
| S-10 | 1688.6130 | H3N5F1 | 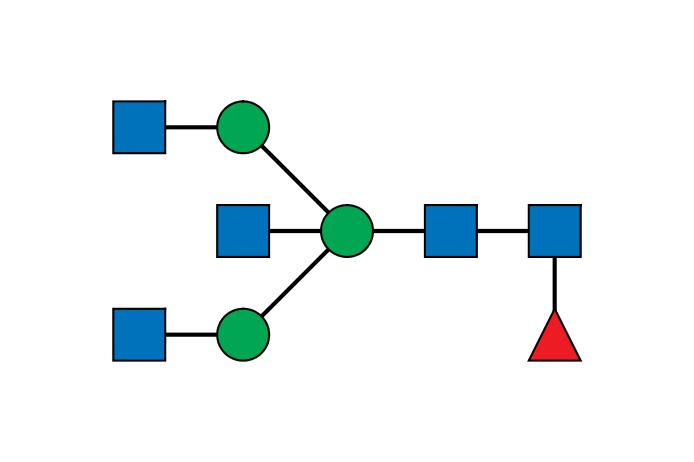 |
| S-11 | 1704.6079 | H4N5 | 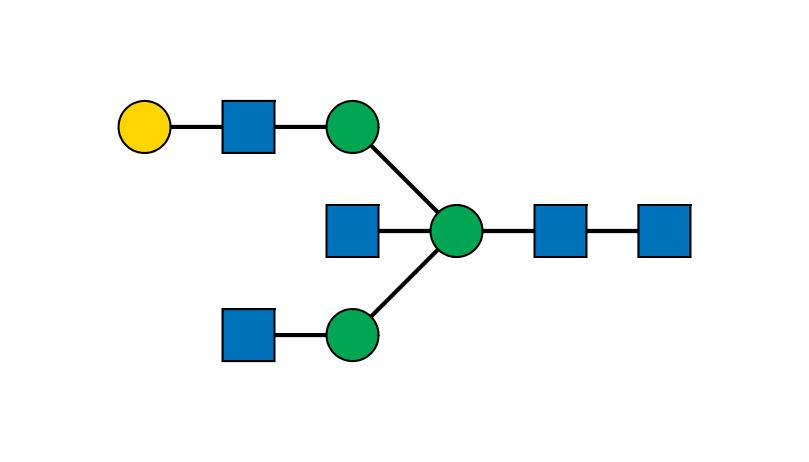 |
| S-12 | 1743.5811 | H8N2 | 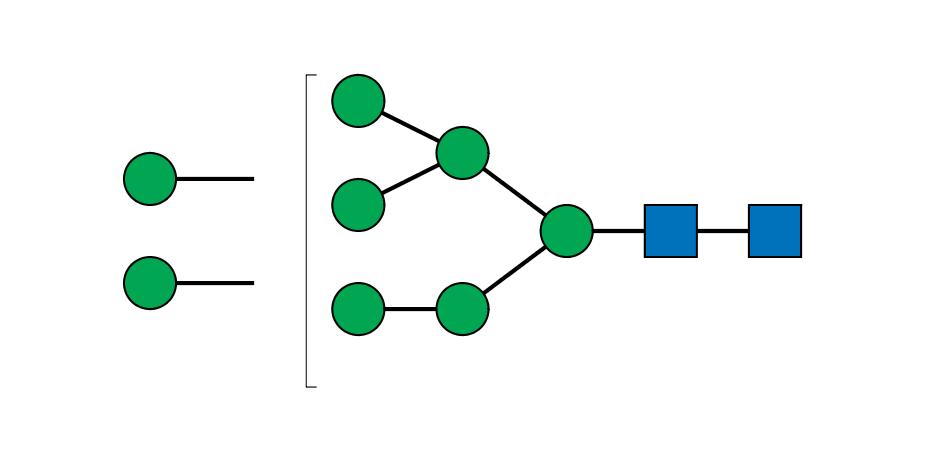 |
| S-13 | 1809.6393 | H5N4F1 | 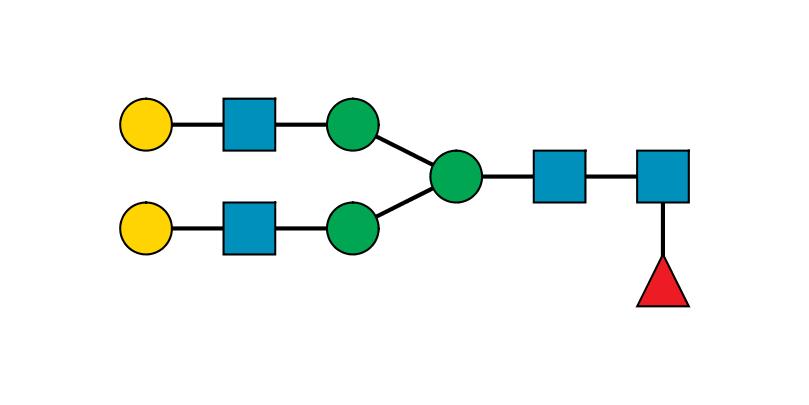 |
| S-14 | 1850.6659 | H4N5F1 | 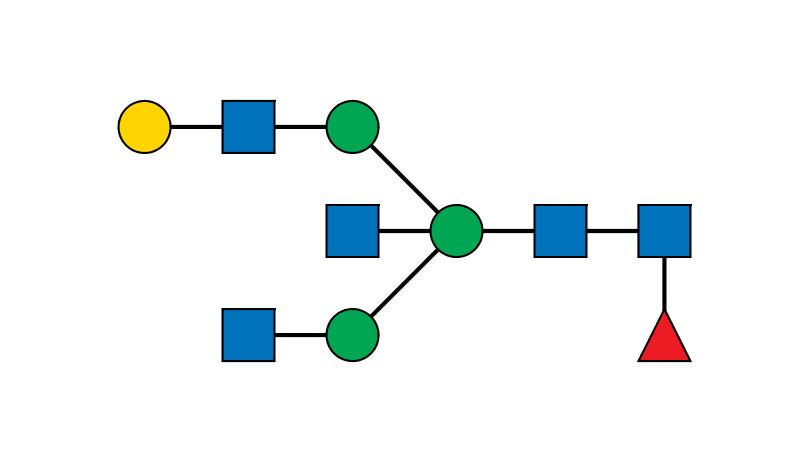 |
| S-15 | 1866.6608 | H5N5 | 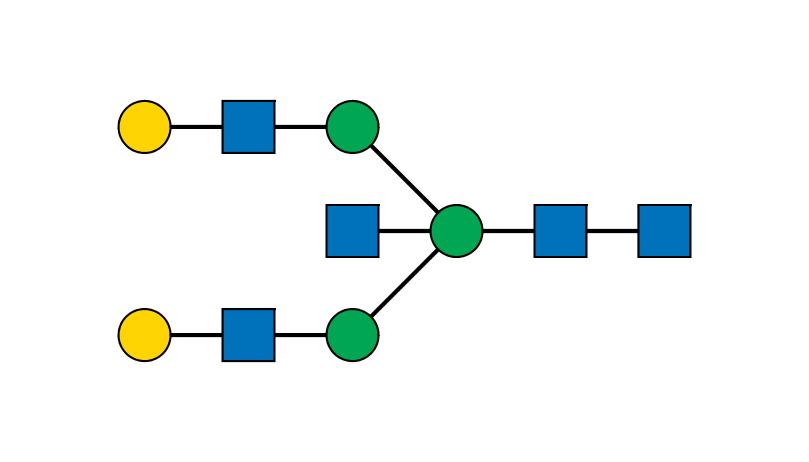 |
| S-16 | 1905.6339 | H9N2 | 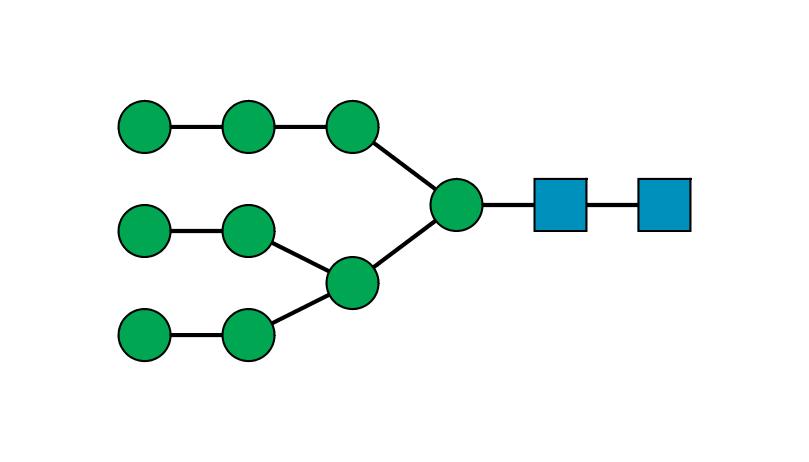 |
| S-17 | 1951.6819 | H4N4F1S1 | 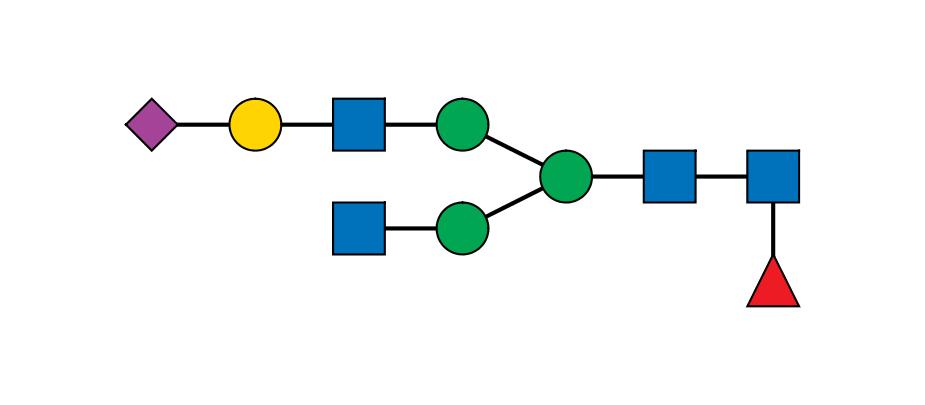 |
| S-18 | 1967.6768 | H5N4S1 | 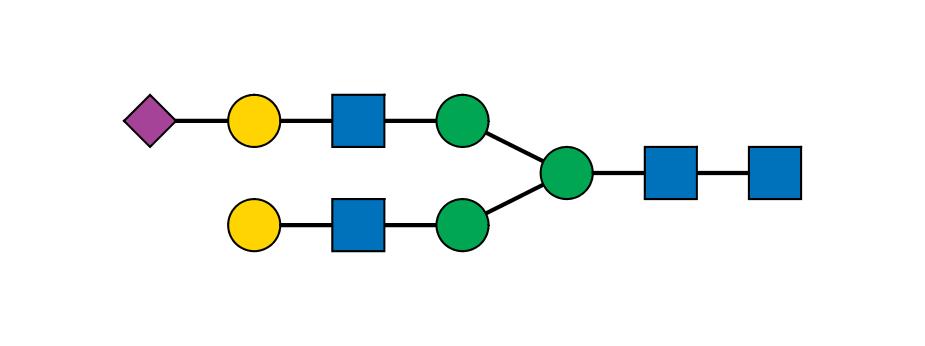 |
| S-19 | 2008.7034 | H4N5S1 | 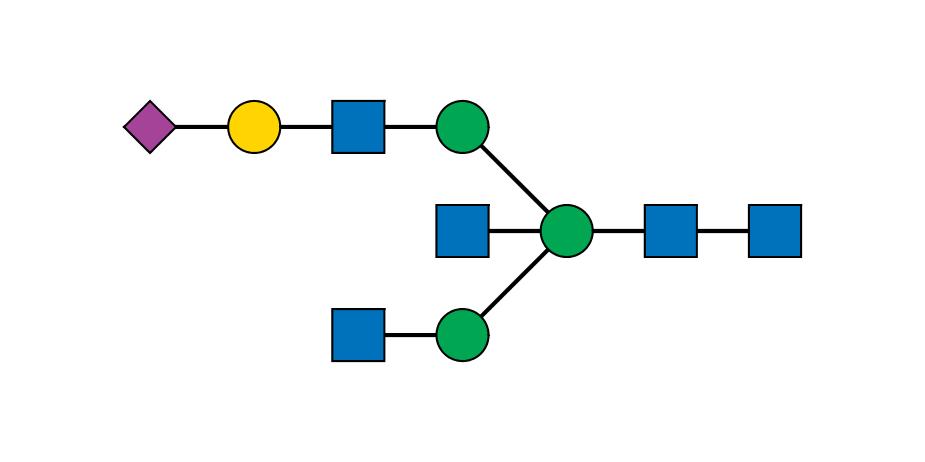 |
| S-20 | 2012.7187 | H5N5F1 | 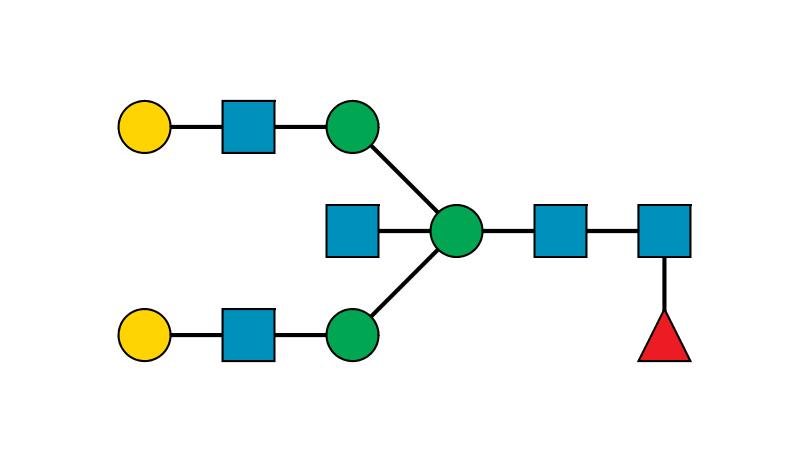 |
| S-21 | 2113.7347 | H5N4F1S1 | 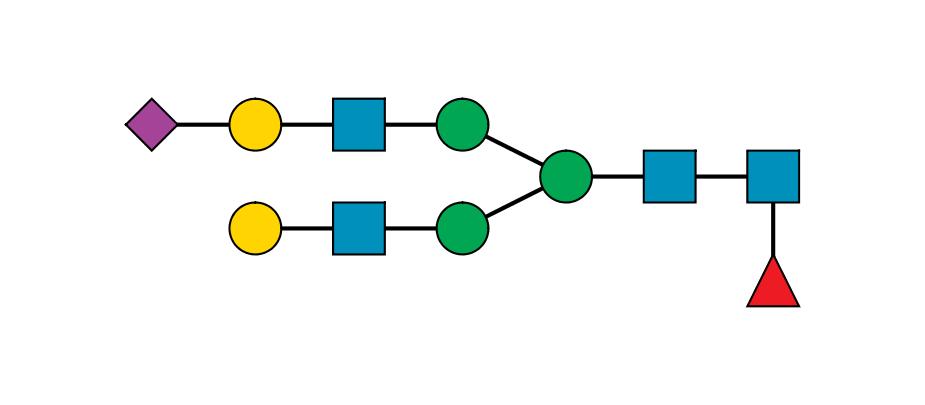 |
| S-22 | 2170.7562 | H5N5S1 | 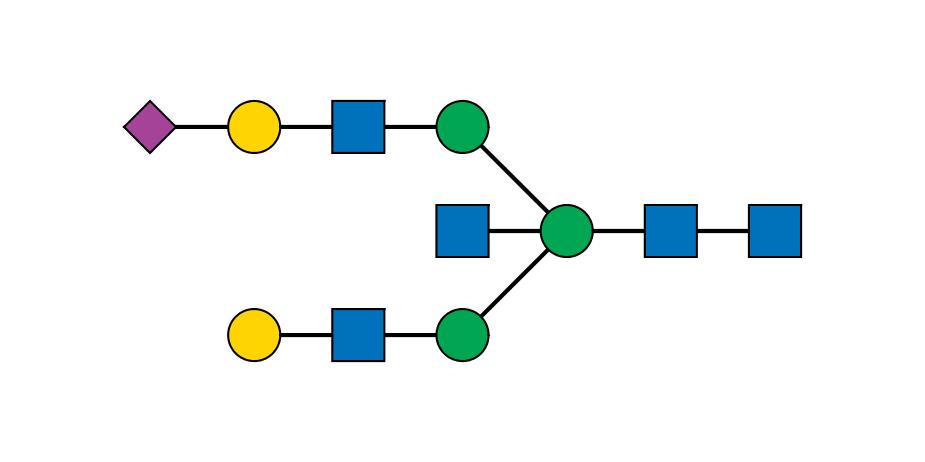 |
| S-23 | 2271.7722 | H5N4S2 | 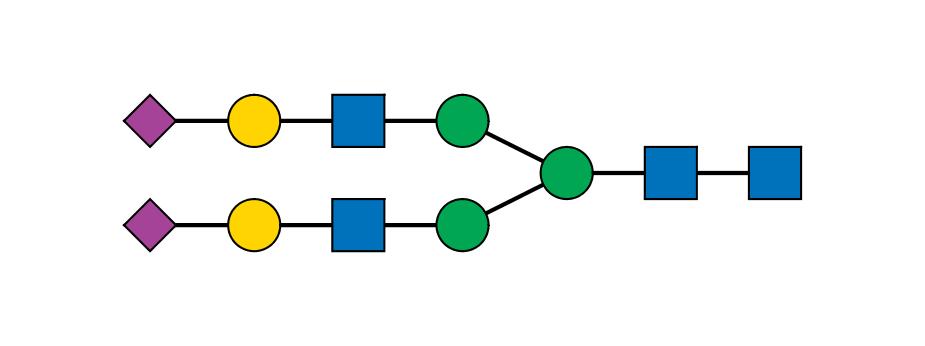 |
| S-24 | 2316.8141 | H5N5F1S1 | 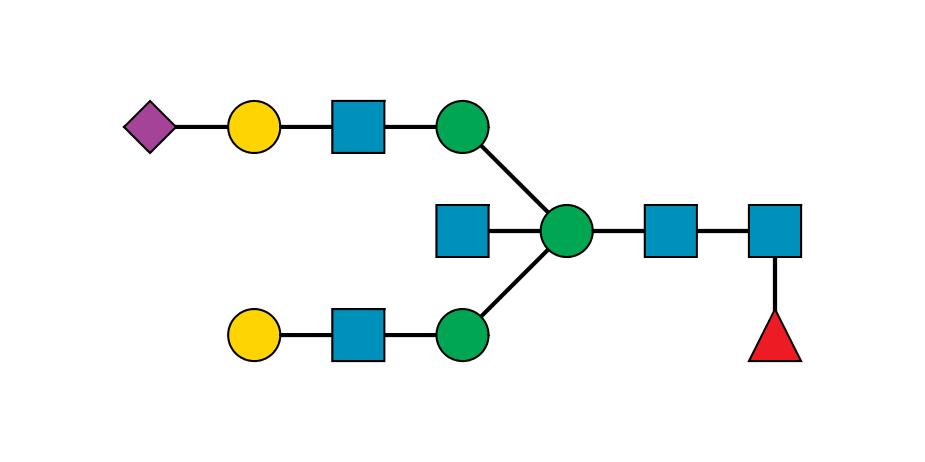 |
| S-25 | 2417.8301 | H5N4F1S2 | 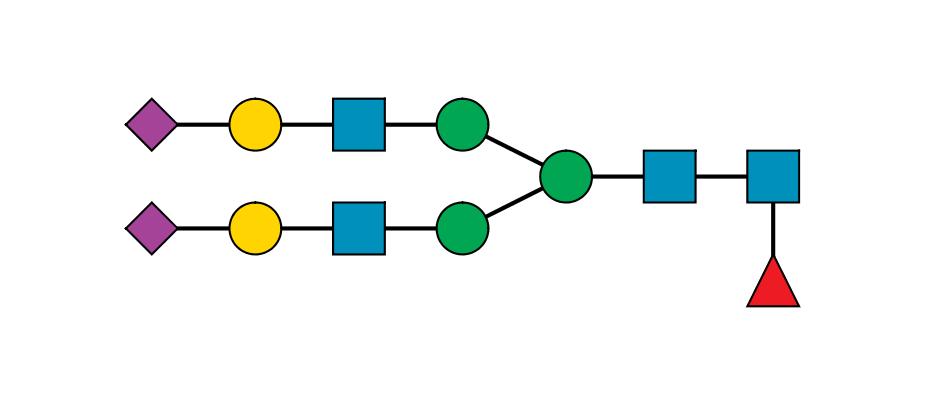 |
| S-26 | 2474.8516 | H5N5S2 | 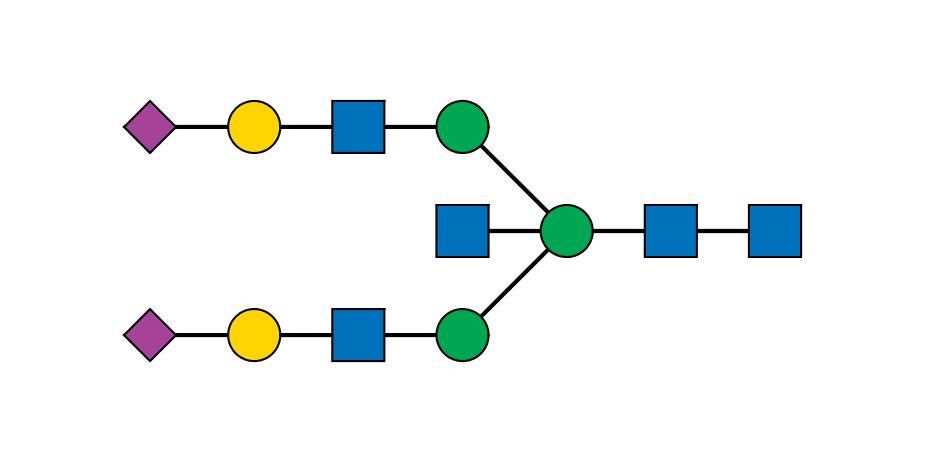 |
| S-27 | 2620.9095 | H5N5F1S2 | 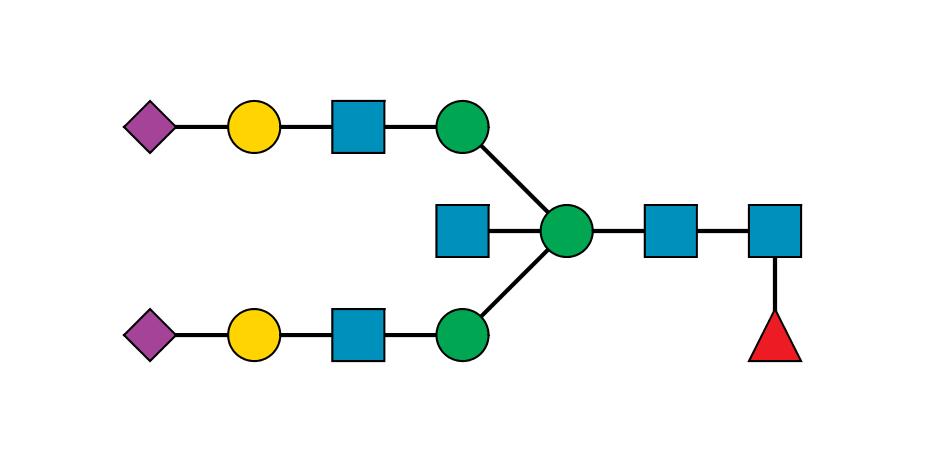 |
| S-28 | 2636.9044 | H6N5S2 | 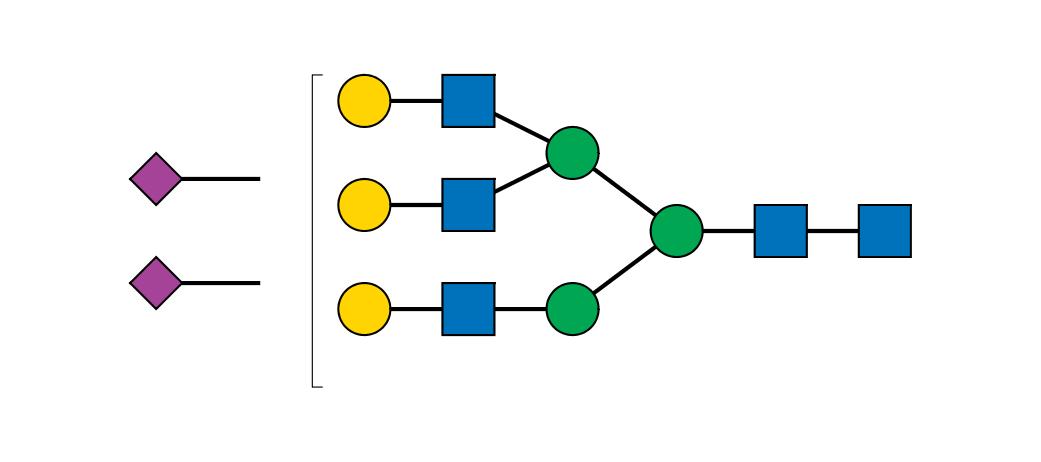 |
| S-29 | 2782.9623 | H6N5F1S2 | 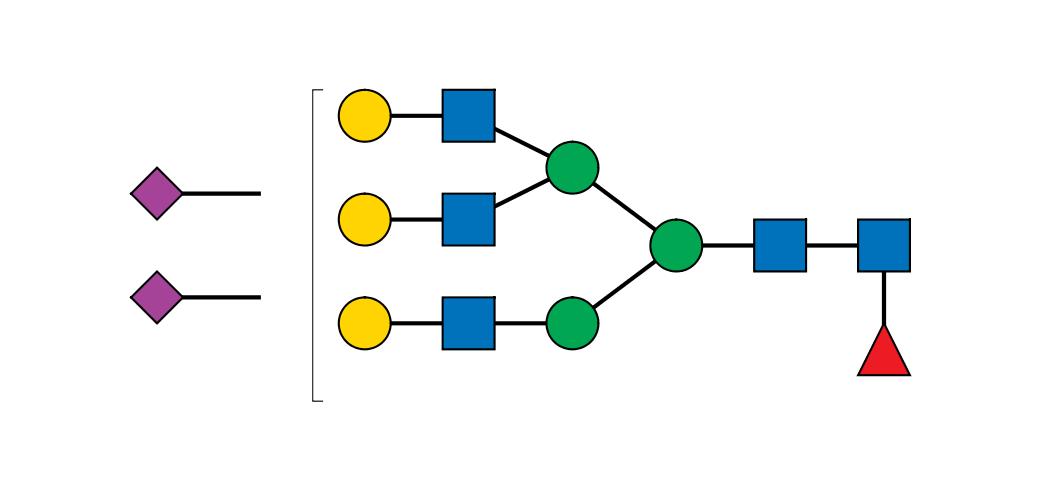 |
| S-30 | 2940.9998 | H6N5S3 | 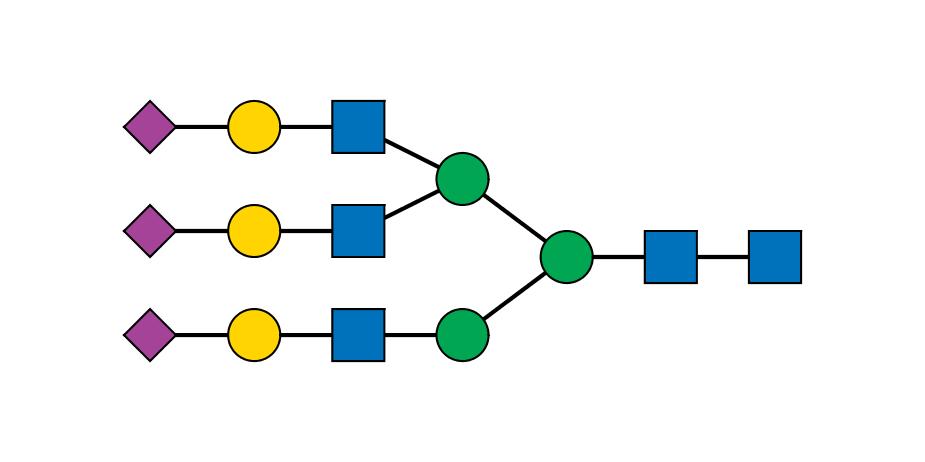 |
| S-31 | 3087.0577 | H6N5F1S3 | 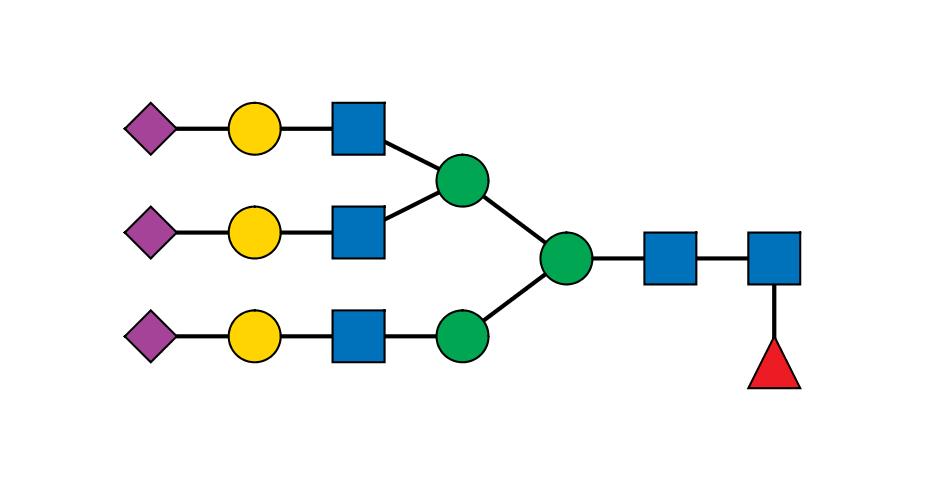 |

*Abbreviations and symbols used for N-glycans composition were as follows: Hexose (H) includes mannose (green circle) and galactose (yellow circle), N-acetylhexosamine (N) includes N-acetylglucosamine (blue square) and N-acetylgalactosamine (yellow square), N-acetylneuraminic acid (S, pink diamond) and fucose (F, red triangle). The different types of glycogroups associated with individual N-glycan compositions are distinguished numerically. Oxford annotation was used for glycan structure proposition.*

**Table S10.** Normal distribution for N-glycome from serum of human

| **N-glycan ions (S)** | **Chemical composition** | **Group** | ***p* value (I vs N) ^a^** |
| --- | --- | --- | --- |
| S-1 | H5N2 | Control | 0.174 |
|  |  | Ischemia | 0.116 |
| S-2 | H4N3 | Control | < 0.001 |
|  |  | Ischemia | < 0.001 |
| S-3 | H3N4 | Control | 0.077 |
|  |  | Ischemia | 0.099 |
| S-4 | H6N2 | Control | 0.028 |
|  |  | Ischemia | 0.006 |
| S-5 | H3N4F1 | Control | 0.541 |
|  |  | Ischemia | 0.124 |
| S-6 | H4N4 | Control | < 0.001 |
|  |  | Ischemia | 0.003 |
| S-7 | H3N5 | Control | 0.145 |
|  |  | Ischemia | 0.310 |
| S-8 | H4N4F1 | Control | 0.410 |
|  |  | Ischemia | 0.434 |
| S-9 | H5N4 | Control | < 0.001 |
|  |  | Ischemia | 0.003 |
| S-10 | H3N5F1 | Control | 0.510 |
|  |  | Ischemia | 0.903 |
| S-11 | H4N5 | Control | 0.105 |
|  |  | Ischemia | 0.111 |
| S-12 | H8N2 | Control | < 0.001 |
|  |  | Ischemia | < 0.001 |
| S-13 | H5N4F1 | Control | 0.488 |
|  |  | Ischemia | 0.077 |
| S-14 | H4N5F1 | Control | 0.208 |
|  |  | Ischemia | 0.289 |
| S-15 | H5N5 | Control | 0.534 |
|  |  | Ischemia | 0.970 |
| S-16 | H9N2 | Control | < 0.001 |
|  |  | Ischemia | < 0.001 |
| S-17 | H4N4F1S1 | Control | 0.075 |
|  |  | Ischemia | 0.058 |
| S-18 | H5N4S1 | Control | 0.080 |
|  |  | Ischemia | 0.811 |
| S-19 | H4N5S1 | Control | < 0.001 |
|  |  | Ischemia | < 0.001 |
| S-20 | H5N5F1 | Control | 0.605 |
|  |  | Ischemia | 0.666 |
| S-21 | H5N4F1S1 | Control | 0.879 |
|  |  | Ischemia | 0.086 |
| S-22 | H5N5S1 | Control | 0.370 |
|  |  | Ischemia | 0.490 |
| S-23 | H5N4S2 | Control | 0.642 |
|  |  | Ischemia | 0.181 |
| S-24 | H5N5F1S1 | Control | 0.263 |
|  |  | Ischemia | 0.196 |
| S-25 | H5N4F1S2 | Control | 0.187 |
|  |  | Ischemia | 0.703 |
| S-26 | H5N5S2 | Control | < 0.001 |
|  |  | Ischemia | < 0.001 |
| S-27 | H5N5F1S2 | Control | 0.110 |
|  |  | Ischemia | 0.078 |
| S-28 | H6N5S2 | Control | | < 0.001 | | --- | |
|  |  | Ischemia | 0.016 |
| S-29 | H6N5F1S2 | Control | < 0.001 |
|  |  | Ischemia | < 0.001 |
| S-30 | H6N5S3 | Control | 0.960 |
|  |  | Ischemia | 0.088 |
| S-31 | H6N5F1S3 | Control | < 0.001 |
|  |  | Ischemia | 0.007 |

***^a^****Shapiro-Wilk test with a confidence interval of 95% was used to assess the normal distribution.*

**Table S11.** Homogeneity of variance for N-glycans from control and ischemic serum of human

| **N-glycan ions (S)** | **Chemical composition** | ***p* value ^a^** |
| --- | --- | --- |
| S-1 | H5N2 | 0.002 |
| S-3 | H3N4 | 0.029 |
| S-5 | H3N4F1 | 0.150 |
| S-7 | H3N5 | 0.031 |
| S-8 | H4N4F1 | 0.064 |
| S-10 | H3N5F1 | 0.114 |
| S-11 | H4N5 | 0.307 |
| S-13 | H5N4F1 | 0.689 |
| S-14 | H4N5F1 | 0.01 |
| S-15 | H5N5 | 0.463 |
| S-17 | H4N4F1S1 | 0.402 |
| S-18 | H5N4S1 | 0.429 |
| S-20 | H5N5F1 | 0.464 |
| S-21 | H5N4F1S1 | 0.965 |
| S-22 | H5N5S1 | 0.642 |
| S-23 | H5N4S2 | 0.148 |
| S-24 | H5N5F1S1 | 0.303 |
| S-25 | H5N4F1S2 | 0.074 |
| S-27 | H5N5F1S2 | 0.171 |
| S-30 | H6N5S3 | 0.002 |

***^a^****Levene test with 95 CI was used to identify the homogeneity of variance.*

**Table S12.** Significantly changed N-glycans from human sera of control and ischemic samples

| **N-glycan (S)** | **Chemical composition** | **Control (C, n=35)** | **Ischemia (I, n=24)** | ***p* value** | ***Adjusted p value ^c^*** |
| --- | --- | --- | --- | --- | --- |
| S-8 | H4N4F1 | 8.314 ± 1.860 | 6.689 ± 2.597 | 0.007 ^a^ | 0.021 |
| S-13 | H5N4F1 | 2.677 ± 0.900 | 2.178 ± 0.859 | 0.037 ^a^ | 0.056 |
| S-21 | H5N4F1S1 | 3.678 ± 0.952 | 2.942 ± 0.948 | 0.005 ^a^ | 0.030 |
| S-23 | H5N4S2 | 46.169 ± 4.662 | 49.005 ± 5.724 | 0.041 ^a^ | 0.049 |
| S-25 | H5N4F1S2 | 3.100 ± 1.113 | 2.384 ± 0.794 | 0.009 ^a^ | 0.018 |
| S-30 | H6N5S3 | 2.630 ± 0.887 | 3.228 ± 1.286 | 0.050 ^b^ | 0.050 |

***^a^****Two-tailed T-test with 95 CI was used when the variance was equal.* ***^b^*** *Two-tailed Mann-Whitney U test with 95 CI was used when the variance was unequal.* ***^c^*** *FDR correction used Benjamini-Hochberg test.*

Table S13. Effect sizes of significantly changed N-glycans and glycosylation features from human serum between control and ischemic groups

| **N-glycan & Glycosylation** | **Chemical**  **composition** | **Confidence interval of 95 % (95 CI)** | **Cohen’s d**  **(C vs I)** |
| --- | --- | --- | --- |
| S-8 | H4N4F1 | -2.787 to -0.465 | 0.368 |
| S-13 | H5N4F1 | -0.968 to -0.030 | 0.280 |
| S-21 | H5N4F1S1 | -1.240 to -0.232 | 0.383 |
| S-23 | H5N4S2 | 0.120 to -5.551 | 0.274 |
| S-25 | H5N4F1S2 | -1.245 to -0.187 | 0.356 |
| S-30 | H6N5S3 | 0.031 to 1.163 | 0.278 |
| HS-M | Mannosylation | -0.346 to 0.406 | 0.031 |
| HS-F | Fucosylation | -7.561 to -1.151 | 0.357 |
| HS-S | Sialylation | -0.535 to -5.244 | 0.215 |

**Table S14.** Significantly changed N-glycans from human serum between control and different ischemic stage groups

| **N-glycan (S)** | **Chemical composition** | ***p* value (C vs T)** | ***p* value (C vs O)** | ***p* value (T vs O)** |
| --- | --- | --- | --- | --- |
| S-3^b^ | H3N4 | 0.602 | 0.037 | 0.012 |
| S-4^a^ | H6N2 | 0.039 | 0.916 | 0.118 |
| S-5^a^ | H3N4F1 | 0.025 | 0.763 | 0.042 |
| S-6^b^ | H4N4 | 0.121 | 0.169 | 0.033 |
| S-8^a^ | H4N4F1 | < 0.001 | 0.472 | 0.022 |
| S-10^a^ | H3N5F1 | 0.506 | 0.013 | 0.009 |
| S-13^a^ | H5N4F1 | 0.016 | 0.408 | 0.214 |
| S-15^a^ | H5N5 | 0.022 | 0.827 | 0.045 |
| S-18^a^ | H5N4S1 | 0.260 | 0.099 | 0.024 |
| S-21^a^ | H5N4F1S1 | 0.001 | 0.223 | 0.111 |
| S-22^a^ | H5N5S1 | 0.030 | 0.892 | 0.105 |
| S-23^a^ | H5N4S2 | 0.009 | 0.596 | 0.093 |
| S-25^a^ | H5N4F1S2 | 0.048 | 0.028 | 0.767 |
| S-26^a^ | H5N5S2 | 0.559 | 0.012 | 0.088 |
| S-30^b^ | H6N5S3 | 0.039 | 0.999 | 0.137 |

***^a^****ANOVA analysis with LSD test was used when the variance was equal.* ***^b^****ANOVA analysis with Tamhane T2 test was used when the variance was unequal. NS, no significance* *statistically.*

**Table S15.** Glycosylation types of detected glycans from human serum of control and ischemic samples

| **N-glycans type** | ***p* value (C vs I)** | ***p* value (C vs T) ^c^** | ***p* value (C vs O) ^c^** | ***p* value (T vs O) ^c^** |
| --- | --- | --- | --- | --- |
| HS-M | 0.153 ^a^ | 0.039 | 0.916 | 0.118 |
| HS-F | 0.009 ^b^ | < 0.001 | 0.452 | 0.016 |
| HS-S | 0.143 ^b^ | 0.003 | 0.552 | 0.005 |

***^a^****Two-tailed T-test with 95 CI was used when the variance was equal.* ***^b^*** *Two-tailed Mann-Whitney U test with 95 CI was used when the variance was unequal.* ***^c^****ANOVA analysis with LSD test was used when the variance was equal.*

*HS-M, mannosylation of detected glycans. HS-F, fucosylation of detected glycans. HS-S, sialylation of detected glycans.*
